# Quality by design (QbD)-guided development of PEGylated nanostructured lipid carriers of donepezil for intranasal delivery

**DOI:** 10.1007/s13346-026-02061-5

**Published:** 2026-02-24

**Authors:** Aneri Desai, Nimeet Desai, Lalitkumar K. Vora, Pranav Shah

**Affiliations:** 1 Maliba Pharmacy College, Uka Tarsadia University, Tarsadi, Bardoli, Gujarat 394350 India; 2 Department of Eye and Vision Science, Institute of Life Course and Medical Sciences, University of Liverpool, Liverpool, L7 8TX UK; 3 School of Pharmacy, Queen’s University Belfast, 97 Lisburn Road, Belfast, BT9 7BL UK

**Keywords:** Quality by Design, Intranasal delivery, PEGylation, Nanostructured lipid carriers, Donepezil

## Abstract

Neurological disorders (ND) pose a major global health challenge, in large part due to the restrictive nature of the blood–brain barrier (BBB), which prevents most therapeutic agents from reaching the central nervous system (CNS). Intranasal delivery (IN) offers a non-invasive and patient-friendly route to bypass the BBB via the olfactory and trigeminal pathways, but its success requires advanced nanocarrier systems capable of enhancing drug retention, stability, and controlled release. In this study, a Quality by Design (QbD) framework was applied to systematically develop and optimize 1,2-distearoyl-sn-glycero-3-phosphoethanolamine (DSPE-PEG_2000_)-functionalized nanostructured lipid carriers (NLCs) using Donepezil (DPZ) as a model drug. Through sequential risk assessment, Plackett–Burman screening design (PBD), and Central Composite Design (CCD) optimization, the Critical Formulation Variables (CFVs) and Critical Process Parameters (CPPs) influencing particle size (PS) and entrapment efficiency (%EE) were identified. The optimized DSPE-PEG_2000_—NLCs exhibited nanoscale size (133.4 ± 2.91 nm), high %EE (89.5 ± 1.51%), strong mucin binding (84.6 ± 2.68%), and a distinct core–shell morphology. In vitro and ex vivo studies confirmed a biphasic and sustained drug release up to 60 h & 67 h, outperforming uncoated NLCs and conventional formulations. Stability studies demonstrated improved preservation under refrigerated conditions. Beyond DPZ, the QbD-guided strategy presented here provides a generalizable and regulatory-aligned platform for designing IN nanocarriers, paving the way for reproducible, scalable, and translational drug delivery systems targeting a wide range of ND.

**Graphical Abstract:**

**Figure:**
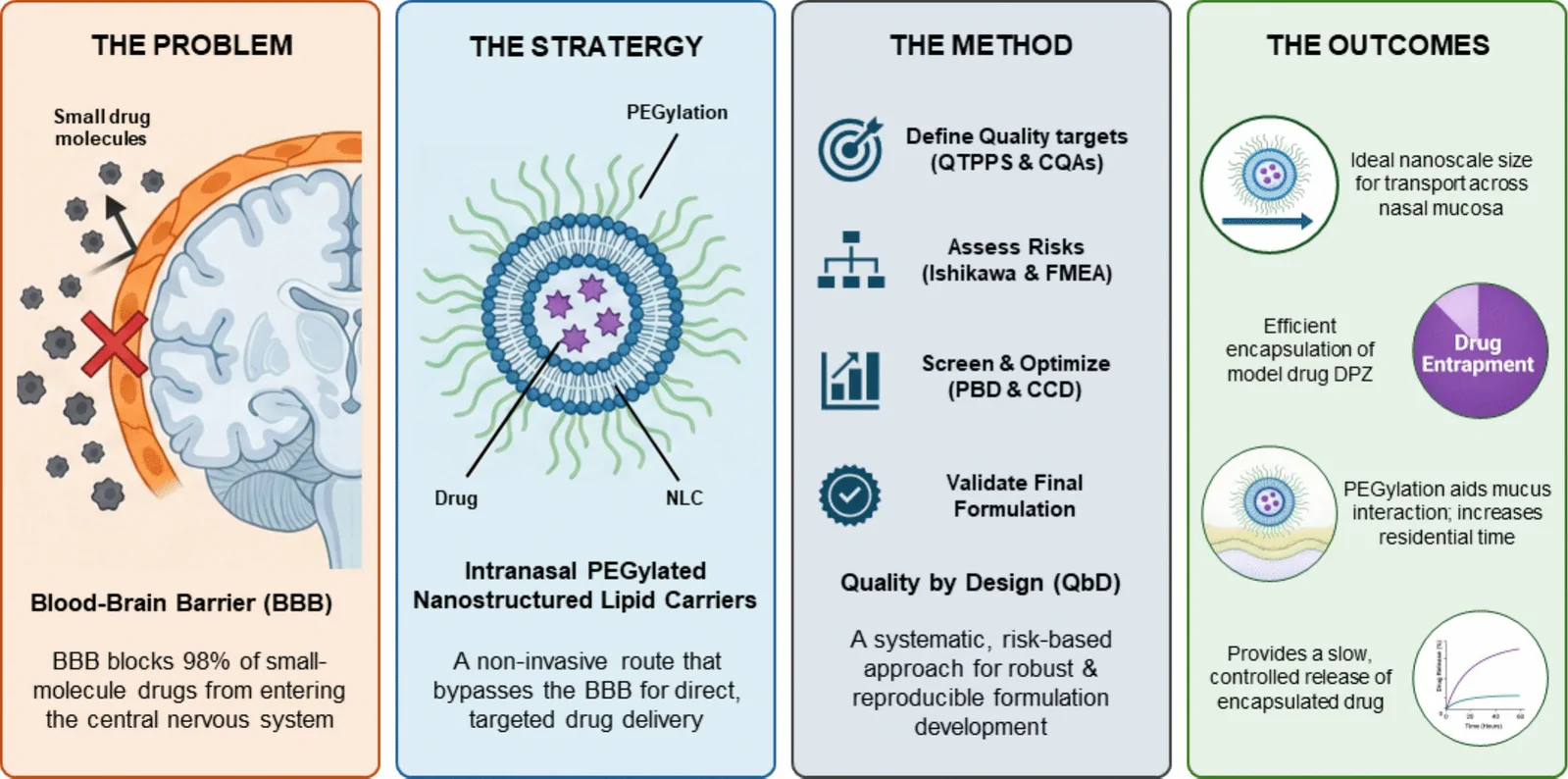

**Supplementary Information:**

The online version contains supplementary material available at https://doi.org/10.1007/s13346-026-02061-5.

## Introduction

Alzheimer’s disease (AD) is a chronic, progressive neurodegenerative disorder characterized by cognitive impairment, memory decline, and reduced ability to perform daily activities. Although its exact cause remains unclear, AD is widely recognized as a multifactorial condition involving cholinergic dysfunction together with the accumulation of amyloid plaques and neurofibrillary tangles in the brain. Currently, four drugs are approved for symptomatic management of AD: the NMDA receptor antagonist memantine, and three acetylcholinesterase (AChE) inhibitors—rivastigmine, donepezil (DPZ), and galantamine [1]. DPZ is a non-competitive and reversible AChE inhibitor with high selectivity in both target and tissue sites. It’s a second approved drug by the United states food and drug administration (USFDA) for the treatment of AD. Its primary pharmacological action is enhancement of cholinergic transmission; however, additional neuroprotective effects have also been reported, including attenuation of β-amyloid–induced neurotoxicity, prevention of loss of nicotinic binding, and reduction of glutamate-induced neurotoxicity [2]. Owing to these properties, DPZ is widely prescribed for patients with mild to moderate AD, where it helps improve cognitive performance and reduce neuropsychiatric symptoms.

DPZ is commercially marketed mainly as oral tablets (5, 10, and 23 mg) [3]. However, oral delivery is associated with several drawbacks, including extensive first-pass metabolism, gastrointestinal adverse effects, and limited brain availability due to restricted penetration across the blood–brain barrier (BBB). Moreover, conventional oral dosage forms provide non-targeted systemic exposure, which contributes to side effects including nausea, diarrhoea, gastric irritation, anorexia, and muscle cramps. Therefore, developing an alternative, non-oral delivery system for DPZ is desirable to minimize gastrointestinal toxicity, reduce systemic distribution to non-target tissues, and improve drug delivery to the brain. Since the therapeutic site of action is in the brain, the selected delivery strategy should be able to achieve effective drug concentrations in brain tissue. In this context, the intranasal (IN) route has been considered a promising approach for targeting DPZ directly to the brain while circumventing the limitations associated with oral administration [4].

Drugs administered intranasally can exploit the unique anatomical connections of the olfactory and trigeminal nerves, enabling direct access to the CNS while largely avoiding first-pass metabolism. The IN route offers the advantages of rapid absorption, reduced pre-systemic metabolism, avoid non-specific side effects, potential for self-administration, and improved patient compliance [5, 6]. To address the issue of oral administration and conventional treatments, the IN route has been explored by a few research groups. Bhava et al. prepared DPZ-chitosan nanosuspension by the ionic crosslinking method and evaluated for an in vitro and ex vivo permeation study [7]. Yasir et al. developed DPZ-loaded solid lipid nanoparticles (SLNs) for IN delivery, where they employed a Box-Behnken design for optimization [4]. Espinoza et al. prepared DPZ-loaded nanoemulsions with Pluronic F-127 and evaluated them for in vitro and ex vivo studies [8]. Topal et al. prepared DPZ-loaded SLNs and targeted them with apolipoprotein E [9].

Nevertheless, rapid mucociliary clearance, limited epithelial permeability, and enzymatic degradation can reduce the drug transport through the IN route, as the formulation is quickly removed from the nasal cavity, resulting in a short residence time [10]. Additionally, hydrophilic and high-molecular-weight compounds still face limited permeability across the epithelial barrier [11]. Overcoming these barriers requires specialised delivery systems capable of protecting the drug, prolonging mucosal residence, and facilitating controlled release [10, 12]. DPZ's hydrophobic nature and low water solubility (0.0171 mg/mL) make it well-suited for a lipid-based system. Among lipid-based nanocarriers, Nanostructured Lipid Carriers (NLCs), a second-generation SLNs, offer superior entrapment efficiency (%EE), enhanced drug loading capacity (%DL), minimal drug leakage during storage, and ER profile compared to other colloidal carriers such as SLNs, liposomes, and emulsion systems [13–15]. Additionally, the lipids used in NLCs are more cost-effective than the phospholipids used in liposome preparation [16]. Their PS typically falls below 200 nm, which is favourable for nose-to-brain transport through both paracellular and transcellular pathways [17, 18].

The physical and chemical properties of nanoparticles influence their pharmacokinetics and biodistribution. For instance, the size of nanoparticles and their surface charge could increase protein binding, resulting in NP uptake and internalization by macrophages, known as the reticuloendothelial system. To enhance the blood half-life of the NP, scientists have used methods to modify or functionalize the surface of nanoparticles. For this purpose, Surface functionalisation with DSPE-PEG_2000_ is a popular method that has been widely used by researchers to enhance the utility of NLCs for IN delivery [12].

In the IN context, PEGylation confers additional benefits by improving interaction with the mucosal environment by reducing mucociliary clearance, enhancing mucin binding efficiency (%MB), and increasing residence time in the nasal cavity [12]. PEGylated lipid-based nanoparticles have been shown to display improved pharmacokinetics, biodistribution, and brain uptake in several preclinical studies, supporting their potential as a robust IN delivery platform [19–22]. To date, relatively few studies have examined PEGylated NLCs in brain-related disorders. Wang et al. developed PEGylated NLCs loaded with biochanin A and reported a smaller particle size (PS), high %EE, and a biphasic drug-release profile [23]. Taha et al. formulated PEGylated liposomes encapsulating verapamil for IN delivery to overcome mucociliary clearance and thereby reduce drug loss within the nasal cavity [24]. Similarly, Mihoub et al. prepared curcumin-loaded PEGylated nanoliposomes for IN administration, demonstrating enhanced neuroprotective effects through the reduction of Aβ plaque formation in AD [25]. Most notably, these studies predominantly relied on empirical or trial-and-error formulation strategies. In contrast, structured methodologies such as Quality by Design (QbD), statistical design of experiments (DoE), and risk-based assessments provide a science-driven framework that allows for simultaneous evaluation of formulation variables (for example, lipid ratios and surfactant concentrations) along with process parameters (such as homogenisation speed, homogenisation time, and stirring time), resulting in improved robustness and scalability.

QbD provides a comprehensive, science- and risk-based approach to formulation development that addresses these gaps [26]. Endorsed by the US FDA and EMA, QbD involves the systematic identification of a Quality Target Product Profile (QTPP), the determination of CQAs, the identification of CPPs, and Critical Material Attributes (CMAs) [27]. Using statistical and DoE tools such as PBD for screening and CCD for optimization, QbD ensures robustness, reproducibility, and regulatory alignment [28]. By proactively assessing risk through tools like Ishikawa diagrams and Failure Mode and Effects Analysis (FMEA), QbD not only reduces the likelihood of batch failures but also streamlines the translation of laboratory formulations into scalable manufacturing processes [29].

Only a few researchers have adopted the QbD approach for the systematic screening and optimization of lipid-based nanocarriers. Sarwar et al. developed olmesartan-loaded NLCs using a QbD framework, incorporating preliminary risk assessment through a fishbone diagram, followed by optimization using a central composite design (CCD) [30]. Similarly, Nagarjun et al. formulated ibrutinib-loaded NLCs, employing Plackett–Burman design (PBD) for factor screening and CCD for optimization [31]. Harsh et al. also applied a systematic QbD strategy to erlotinib-loaded chitosan nanoparticles, utilizing Taguchi design for initial screening and CCD for further optimization [32].

In the context of DPZ’s, previous IN formulations have primarily involved SLNs [9], microemulsions [33, 34], and gels/hydrogels [35, 36]. Although some studies reported improved brain bioavailability but they lacked systematic formulation screening, did not employ PEGylation, and provided limited evaluation of Critical Quality Attributes (CQAs) such as PS, %EE, and stability. Overall, none of the existing studies have integrated a comprehensive QbD-based development strategy alongside extensive physicochemical and performance characterization for IN PEGylated NLC systems. By focusing on the methodological framework rather than a single disease indication, this study establishes a reproducible and regulatory-aligned strategy for the rational design of PEGylated lipid nanocarriers. While DPZ was chosen as a model drug due to its poor solubility and limited brain bioavailability, the framework developed here has broader implications for nose-to-brain delivery of therapeutic agents in diverse ND.

So, the present study aims to develop DPZ-loaded PEGylated NLCs with a desired PS and administered via the IN route using a QbD approach to enhance therapeutic effect and permeation with SR of the drug to treat AD with minimum side effects. The novelty of this study includes i) development of DPZ-PEGylated NLCs, ii) unlike previous DSPE-PEG-based IN formulations that rely predominantly on empirical optimization, this work represents the first comprehensive application of combining sequential risk assessment, DoE, screening, and CCD optimization to establish a robust design space for PEGylated NLCs, iii) The study comprehensively combined QbD framework with a mechanistic investigation linking PEG-corona formation with mucin interaction, sustained diffusion, and prolonged nasal residence demonstrating performance advantages over non-PEGylated NLCs and conventional formulations, iv) the integration of in-vitro release profiling, ex-vivo permeation, mucin-binding analysis, morphological characterization, DSC, XRD and stability evaluation provides a coherent translational framework rather than isolated experimental outcomes. None of the researchers has combined this approach. So, in this work, we present the first complete application of a QbD framework to the development of DPZ-PEGylated NLCs for IN delivery.

## Materials and methods

### Materials

DPZ was obtained as a gift sample from Alembic Pharmaceuticals Pvt. Ltd. (Vadodara, India). Dynasan^®^ 116 was supplied by Gattefosse India Pvt. Ltd. (Mumbai, India), while castor oil was purchased from Yarrow Chem Pvt. Ltd. (Mumbai, India). Tween^®^ 80, Span^®^ 80, and 1,2-distearoyl-sn-glycero-3-phosphoethanolamine-N [methoxy (polyethylene glycol)-_2000_] (DSPE-PEG_2000_) (M. wt = 852.2 g/mol) were procured from Sigma-Aldrich (Mumbai, India). Porcine mucin (PM) was purchased from Baoman Bio (Shanghai, People’s Republic of China). Dialysis membranes (molecular weight cut-off 12,000–14,000 Da), syringe filters (0.22 µm), and membrane filters were sourced from HI Media Laboratories Pvt. Ltd. (Mumbai, India). All other reagents and solvents were of analytical grade and used without further purification.

### Preparation of DPZ-PEGylated NLCs

DPZ-PEGylated NLCs were prepared by a high-speed homogenization method with minor modifications [12, 37]. The lipid phase was composed of DPZ, solid lipid (Dynasan^®^ 116), liquid lipid (castor oil), DSPE-PEG_2000_, and Span^®^ 80, all of which were melted and solubilized at 70 °C. The aqueous phase was prepared separately by dissolving Tween^®^ 80 in Milli-Q water at the same temperature. The preheated surfactant solution was added to the lipid phase under continuous stirring at 1000 rpm, and the resulting hot aqueous dispersion was homogenized using a high-speed homogenizer (Ultra-Turrax T-25, IKA, Germany) at 6200 rpm for 15 min. Non-PEGylated (Uncoated) NLCs were prepared following the same procedure but without DSPE-PEG_2000_. Blank formulations (without drug) were produced identically, excluding both DPZ and DSPE-PEG_2000_ from the lipid phase.

### Preliminary screening

A preliminary study is a useful part of making a formulation ready for optimization. It is one of the screening approaches used to identify the critical factors and their optimum ranges for the preparation of PEGylated NLCs that have the greatest impact on the quality of the formulation. Screening of factors is a primary study that provides a detailed outline of the factors that can be further utilized during product optimization. In the preparation of PEGylated NLCs, several formulation and process parameters significantly influence the quality attributes of the final product. Therefore, a preliminary study of PEGylated NLCs was carried out using a QbD-based approach. In the present study, screening of the amount of DSPE-PEG _2000_, solid-to-liquid lipid ratio, concentration of Tween^®^ 80, concentration of Span 80, homogenization speed, homogenization time, and stirring time was performed using suitable experimental methods within the QbD framework, and the results are shown in Table S1**.**

### Defining the QTPPs and CQAs

The first step in the QbD framework for developing DPZ-PEGylated NLCs was the identification of QTPPs and CQAs. QTPPs were established based on regulatory guidelines, literature reports, and the intended therapeutic objectives to ensure the product’s quality, safety, and efficacy. From this framework, CQAs were defined as the critical physicochemical attributes directly influencing product performance. For DPZ-PEGylated NLCs, the key CQAs identified were PS, zeta potential (ZP), %EE, and in vitro drug release, of which the most significant parameters were prioritised for subsequent risk assessment and optimization [38].

### Initial risk assessment

A preliminary risk assessment was performed to identify the CPPs, CFVs, and CMAs influencing the CQAs of DPZ-PEGylated NLCs. The relationships between these factors were first mapped using an Ishikawa cause–effect diagram generated in Minitab^®^ 22.1.0 (Minitab Inc., PA, USA), linking CMAs (DPZ, Dynasan^®^ 116, castor oil, DSPE-PEG_2000_, Tween^®^ 80, Span^®^ 80), CFVs (solid lipid: liquid lipid ratio, amount of DSPE PEG_2000_, concentration of Tween^®^ 80, concentration of Span^®^ 80), and CPPs (rate of addition, stirring speed, homogenization speed, homogenization time, temperature, and stirring time) to the selected CQAs. Risk was then evaluated qualitatively using a risk estimation matrix (REM) and quantitatively through FMEA. Each parameter was scored for occurrence (O), severity (S), and detectability (D) on a 1–5 scale, where 1 indicated minimal risk and 5 indicated maximum risk. The risk priority number (RPN) was calculated as the product of O × S × D, and a threshold value of ≥ 25 was used to classify parameters as high risk. Factors identified as high risk were prioritised for further screening and included in the PBD [39].

### Screening of significant factors by PBD

To identify the most influential factors affecting the CQAs of DPZ-PEGylated NLCs, a PBD was employed. This two-level fractional factorial design is well-suited for screening a large number of factors with a limited number of experimental runs. Seven factors, representing both CFVs and CPPs, were investigated: solid lipid: liquid lipid ratio, amount of DSPE PEG_2000_, concentration of Tween^®^ 80, concentration of Span^®^ 80, homogenization speed, homogenization time, and stirring time. The responses selected were PS (Y_1_) and %EE (Y_2_). A total of fifteen experimental runs, including three centre points, were generated using Design-Expert^®^ 13.0 software (Stat-Ease Inc., Minneapolis, MN, USA) [40]. The resulting data were analysed using ANOVA to determine the statistical significance of each factor. Pareto chart analysis further illustrated the relative contribution of individual material attributes, formulation variables, and process parameters to the responses. Based on the PBD results, the most significant factors were identified and finalized for optimization using CCD [41].

### Evaluation of DPZ-PEGylated NLCs

The mean PS, PDI, and ZP of DPZ-PEGylated NLCs were determined by dynamic light scattering (DLS) and electrophoretic light scattering (ELS) using a Nano-ZS90 analyser (Malvern Instruments Ltd., USA). Samples were diluted tenfold with Milli-Q water and analysed at 25 ± 0.2 °C. Measurements were performed at a 90° scattering angle for PS/PDI and at a 173° scattering angle for ZP. Each sample was measured in triplicate, and results were reported as mean ± SD. %EE of DPZ-NLCs and DPZ-PEGylated NLCs were determined by ultracentrifugation method. Briefly, 10 mL of formulation was centrifuged (REMI C24 Plus, India) at 10,000 rpm for 30 min at 25 °C [4]. The supernatant containing free drug was collected and analysed at λ_max_ = 231 nm using a UV–visible spectrophotometer (Shimadzu 3092, Japan). EE (%) was calculated using the following equation:

1 $$\%EE=\left( \frac{Total\; Drug-Free\; Drug}{Total\; Drug} \right) \times 100$$

### Systematic formulation optimization of DPZ-PEGylated NLCs

A CCD was employed to further analyse the significant parameters identified from PBD. Each factor was evaluated at five levels: low (− 1), high (+ 1), centre (0), and two axial points (− α and + α). A total of nineteen experimental runs were generated by Design-Expert^®^13.0, comprising eight factorial points, six axial points, and five centre points. Formulations were prepared according to the design, and the resulting data were fitted to a second-order polynomial model to evaluate the linear, quadratic, and interaction effects of the independent factors on the responses, PS, and %EE [42]. Statistical significance of the model terms was assessed by ANOVA, with p < 0.05 considered significant. Numerical optimization was carried out using the desirability function, which ranks potential solutions on a scale of 0 to 1, with higher values indicating a closer fit to the target criteria. The formulation with the highest overall desirability was selected as the optimized batch. Graphical optimization was performed using overlay plots to define the design space, where the combination of critical variables satisfied the desired criteria for PS and %EE without violating response constraints. The design was further validated by checkpoint analysis. Experimental results of the optimized formulation were compared with model-predicted values, and the percentage prediction error was calculated to confirm the reliability of the optimization process [43].

### Lyophilisation

The optimized DPZ-PEGylated NLCs dispersion was lyophilised to obtain a dry powder suitable for handling and further characterisation (DSC and XRD). Briefly, 50 mL of the dispersion was mixed with mannitol (3.5% w/v) as a cryoprotectant, transferred into round-bottom flasks, and pre-frozen at − 75 °C for 12 h. Primary drying was performed at 0.1 mbar and − 20 °C for 21 h, followed by secondary drying at 0.010 mbar and 25 °C for 3 h using a freeze dryer (ilShinBioBase CSP-TFD-8503, South Korea) [44].

#### Stability study of lyophilized PEGylated NLCs

The stability study of the optimized freshly prepared freeze-dried DPZ-PEGylated-NLCs was carried out according to the ICH guidelines Q1A(R2). The samples were packed in tightly closed vials and stored at the two different conditions, 5 ± 3 °C (refrigerator), 25 ± 2 °C (room temperature), for 15 days. Samples were analysed on days 0, 3, 6, 9,12 and 15 for PS and %EE to evaluate storage stability under both room temperature and refrigerated conditions, and the data are observed in Table S2**.**

### Characterization of optimized DPZ-PEGylated NLCs

*%EE*

#### PS, PDI, ZP, %EE, and %DL

The PS, PDI, ZP, and %EE of the optimized formulation were determined using the same methods described in "Evaluation of DPZ-PEGylated NLCs". DL (%) was calculated based on the amount of entrapped drug relative to the total formulation weight using the following equation:

2 $$\%DL=\left( \frac{Total\; Drug-Free\; Drug}{Total\; Weight\; of\; NLCs} \right) \times 100$$

#### Drug content (%DC)

One millilitre of optimized formulation was dissolved in 10 mL of methanol, sonicated (Sonica 2400MH S_3_, Italy) for 10 min, and filtered through a 0.45 μm polytetrafluoroethylene membrane (PTFE). The solution was analysed spectrophotometrically at λ_max_ 231 nm, and DC (%) was calculated using the following equation:

3 $$\%Drug\; Content=\left(\frac{Actual\; Drug\; Content}{Theoretical\; Drug\; Content}\right) \times 100$$

#### Surface morphology BY HRTEM

The morphology of uncoated DPZ-NLCs and optimized DPZ-PEGylated NLCs were assessed by HRTEM (ECNAI G2 TF20-ST, FEI, USA) operating at 100 kV. Samples were prepared by adsorbing diluted NLCs onto carbon-coated copper grids, followed by negative staining with 2% w/v phosphotungstic acid. Excess stain was removed, grids were dried, and samples were imaged using a VELETA CCD camera (Olympus Soft Imaging Solutions) [45]. The images provided insight into particle shape, surface smoothness, and DSPE PEG_2000_ coating around NLCs.

#### Differential scanning calorimetry (DSC)

Thermal behaviour was analysed by DSC (Mettler Toledo DSC3, STARe system). Samples (2 mg) were weighed into sealed aluminium pans and scanned from 30 to 340 °C at 10 °C/min under nitrogen flow (10 mL/min). Indium was used for calibration. Thermograms of pure DPZ, mannitol (cryoprotectant), and optimized lyophilized DPZ-PEGylated NLCs were compared to assess drug–excipient interactions and crystalline-to-amorphous transitions [46].

#### Powder X-ray diffraction (XRD)

Crystallinity was assessed by XRD (D8 Advance, Bruker, Germany) using Cu-Kα radiation at 40 kV and 40 mA. Samples were scanned from 10–50° (2θ) with a step size of 0.02° and a scan speed of 4°/min. Diffractograms of pure DPZ, DSPE-PEG_2000,_ and optimized lyophilized DPZ-PEGylated NLCs were compared to evaluate changes in crystalline structure following encapsulation [47].

#### Mucin binding efficiency

The interaction of NLCs with mucin was evaluated by mixing 5 mL of PM (0.5 mg/mL in SNF, pH 6.4) with 5 mL of formulation (optimized DPZ-PEGylated NLCs and uncoated DPZ-NLCs) and incubating at 37 °C for 1 h, followed by centrifugation at 10,000 rpm for 1 h at 4 °C [48]. The experiment was conducted in triplicate. Free mucin in the supernatant was measured at λ_max_ 270 nm, and %MB was calculated using the following equation:

4 $$MB\left(\%\right)=\left( \frac{Total\; Mucin-Free\; Mucin}{Total\; Mucin} \right)\times 100$$

#### In vitro drug release and kinetic modelling

The in vitro release of DPZ from various formulations (DPZ suspension, marketed formulation (Donep^®^), DPZ-NLCs, and optimized DPZ-PEGylated NLCs) was evaluated using a dialysis bag diffusion technique. Briefly, each formulation (5 mL, equivalent to 5 mg DPZ) was placed into a preconditioned dialysis membrane (molecular weight cut-off: 12–14 kDa, regenerated cellulose; Sigma-Aldrich, UK) and sealed at both ends. The bags were immersed in 100 mL of 0.05 M simulated nasal fluid (SNF, pH 6.4) containing 0.1% Tween^®^ 80 to maintain sink conditions. The receptor medium was maintained at 37 ± 0.5 °C under continuous agitation (50 rpm). At predetermined time points, 5 mL samples were withdrawn from the receptor compartment and immediately replaced with equal volumes of fresh medium to maintain constant volume and sink conditions. Drug concentrations were quantified using spectrofluorimetric method (λ_ex_ = 314 nm, λ_em_ = 628 nm; Shimadzu RF-5301 PC, Japan). A validated analytical method was employed; details of linearity, sensitivity (LOD/LOQ), precision, accuracy, and recovery studies are presented in the Supplementary Methods. Cumulative release was calculated after correcting for sample replacement, and data were expressed as mean ± SD (n = 3). To interpret release kinetics, the data were fitted to zero-order, first-order, Higuchi, and Korsmeyer–Peppas models, and the Hixon-Crowell model, and the best-fitting model was selected based on the coefficient of determination (R^2^) [49, 50].

#### Ex vivo drug diffusion study

The ex vivo permeation of DPZ from DPZ-suspension, DPZ-NLCs, and optimized DPZ-PEGylated NLCs was assessed using freshly excised goat nasal mucosa obtained immediately after sacrifice from a local abattoir. The tissue was carefully separated, washed with 0.05 M SNF (pH 6.4), and trimmed to uniform thickness (0.20 ± 0.02 mm) following removal of cartilage. The mucosa was mounted between the donor and receptor chambers of vertical Franz diffusion cells (diffusion area: 1.7 cm^2^; receptor volume: 20 mL), with the epithelial surface facing the donor. The receptor compartment was filled with 0.05 M SNF (pH 6.4, 0.1% Tween^®^ 80) maintained at 37 ± 0.5 °C, and magnetically stirred at 50 rpm [51]. Formulations equivalent to 5 mg DPZ (suspension, DPZ-NLCs, optimized DPZ-PEGylated NLCs) were applied to the donor compartment. At predetermined intervals, 1 mL samples were withdrawn from the receptor and replaced with fresh pre-warmed medium. DPZ concentrations were quantified using the validated spectrofluorimetric method. Cumulative drug permeation was expressed as µg/cm^2^. Steady-state flux (*J *_*ss*_) was calculated from the slope of the linear portion of the permeation curve, and the apparent permeability coefficient (P_app_) was obtained as:

5 $${P}_{app} = \frac{{J}_{ss}}{{C}_{d}}$$

where C_d_ is the donor drug concentration.

#### Storage stability of optimized liquid DPZ-PEGylated NLCs

Stability of the optimized formulation was assessed according to ICH Q1A (R2) guidelines at 25 ± 2 °C and 5 ± 3 °C for 28 days. Samples were analysed on days 0, 7, 14, 21, and 28 for PS and %EE to evaluate storage stability under both room temperature and refrigerated conditions [52].

### Statistical analysis

All experiments were performed in triplicate (n = 3), and results are reported as mean ± standard deviation (SD). Data were analysed using one-way analysis of variance (ANOVA), with p < 0.05 considered statistically significant.

## Results and discussion

### Defining the QTPPs and CQAs

This study aimed to develop DPZ-PEGylated NLCs for IN administration as a potential platform for nose-to-brain delivery in ND. Based on regulatory guidelines and literature reports, QTPPs were defined to ensure the quality, safety, and efficacy of the formulation (Table 1). Key elements included formulation type, route of administration, pharmacokinetic expectations, and stability requirements. PEGylated NLCs were selected as the formulation platform due to their ability to enhance mucoadhesion, reduce mucociliary clearance, prolong nasal residence time, and potentially improve patient compliance. The IN route was chosen to bypass gastrointestinal degradation and hepatic first-pass metabolism associated with conventional oral delivery. From the QTPPs, CQAs were identified and justified. Among them, PS and %EE were prioritised as the most critical. PS plays a dual role: smaller particles (< 200 nm) facilitate transport across the nasal mucosa and enhance potential nose-to-brain delivery, while also contributing to physical stability by reducing aggregation propensity. %EE, defined as the percentage of drug entrapped within the carrier system, directly affects drug payload and dosing efficiency. A higher EE (> 80% w/w) ensures adequate DL, minimizes drug loss, and supports sustained release from the lipid matrix. These parameters were therefore selected as the primary CQAs for subsequent risk assessment and optimization [53].

**Table 1:** QTPPs and CQAs of DPZ-PEGylated NLCs

QTPPs	*Target*	*Justification*
Formulation	PEGylated NLCs	PEGylation reduces mucociliary clearance, prolongs nasal retention, and supports targeted brain delivery
Route of administration	IN	Avoids gastrointestinal degradation and first-pass metabolism associated with oral DPZ
Pharmacokinetics	Improved relative to oral dosage	Targeted nose-to-brain delivery may reduce dose and dosing frequency
Stability	No visible aggregation or caking for at least 1 months	Stability of PS and %EE is critical for sustained formulation performance
**CQAs**	***Target***	***Justification***
PS	Mean < 200 nm	Enhances nasal absorption, improves solubility/dissolution, and reduces aggregation
%EE	Maximum (> 80% w/w)	Ensures higher DL, minimizes drug wastage, and supports sustained release

### Initial risk assessment

**Fig. 1:**
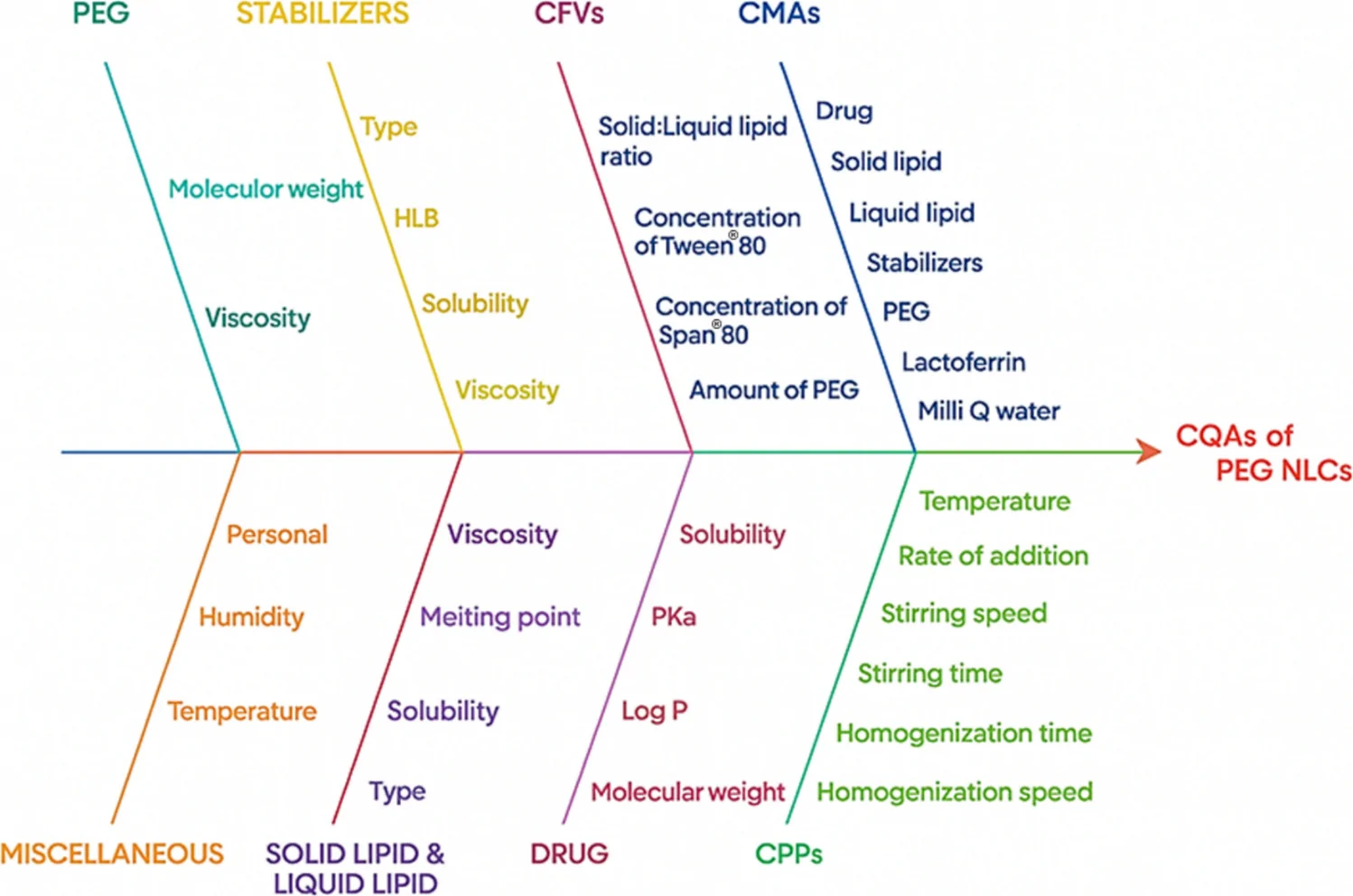
Ishikawa (fishbone) diagram illustrating CMAs, CFVs, and CPPs influencing the CQAs of DPZ-PEGylated NLCs. This systematic mapping supports the QbD-based optimization of DPZ-PEGylated NLCs

An Ishikawa (fishbone) diagram was first constructed to visualise the potential influence of CMAs, CFVs, CPPs, and environmental conditions on the CQAs of DPZ-PEGylated NLCs (Fig. 1). Here, the CQA’s were PS and %EE. Both of these play a major role when administered by IN route. For the targeted drug delivery of NPs to the brain, PS plays a major role in carrying the drug to its targeted site through the olfactory region. Therefore, it was aimed to obtain the PS within < 200 nm. High %EE gives very high drug release at a target site, and to avoid the wastage of drug, thereby increasing the potential of the delivery by increasing the bioavailability of the formulation. Hence, %EE was identified as a CQAs to be optimized for ensuring high EE [54]**.** This qualitative mapping highlighted that both formulation composition and process conditions could significantly affect PS and %EE. Based on preliminary trials, CFVs and CPPs were prioritised for further investigation in the screening design. A REM was subsequently developed to classify risk levels associated with the identified factors (Table 2). From the Ishikawa fishbone diagram, it could be seen that the solid lipid: liquid lipid ratio, concentration of Tween^®^ 80, concentration of Span^®^ 80, amount of DSPE PEG_2000_, homogenization speed, homogenization time, and stirring time represented the highest-risk factors with respect to PS and %EE, which are to be considered for further screening and optimization. In contrast, parameters such as temperature, stirring speed, and rate of addition were categorised as medium to low risk [55].

**Table 2:**
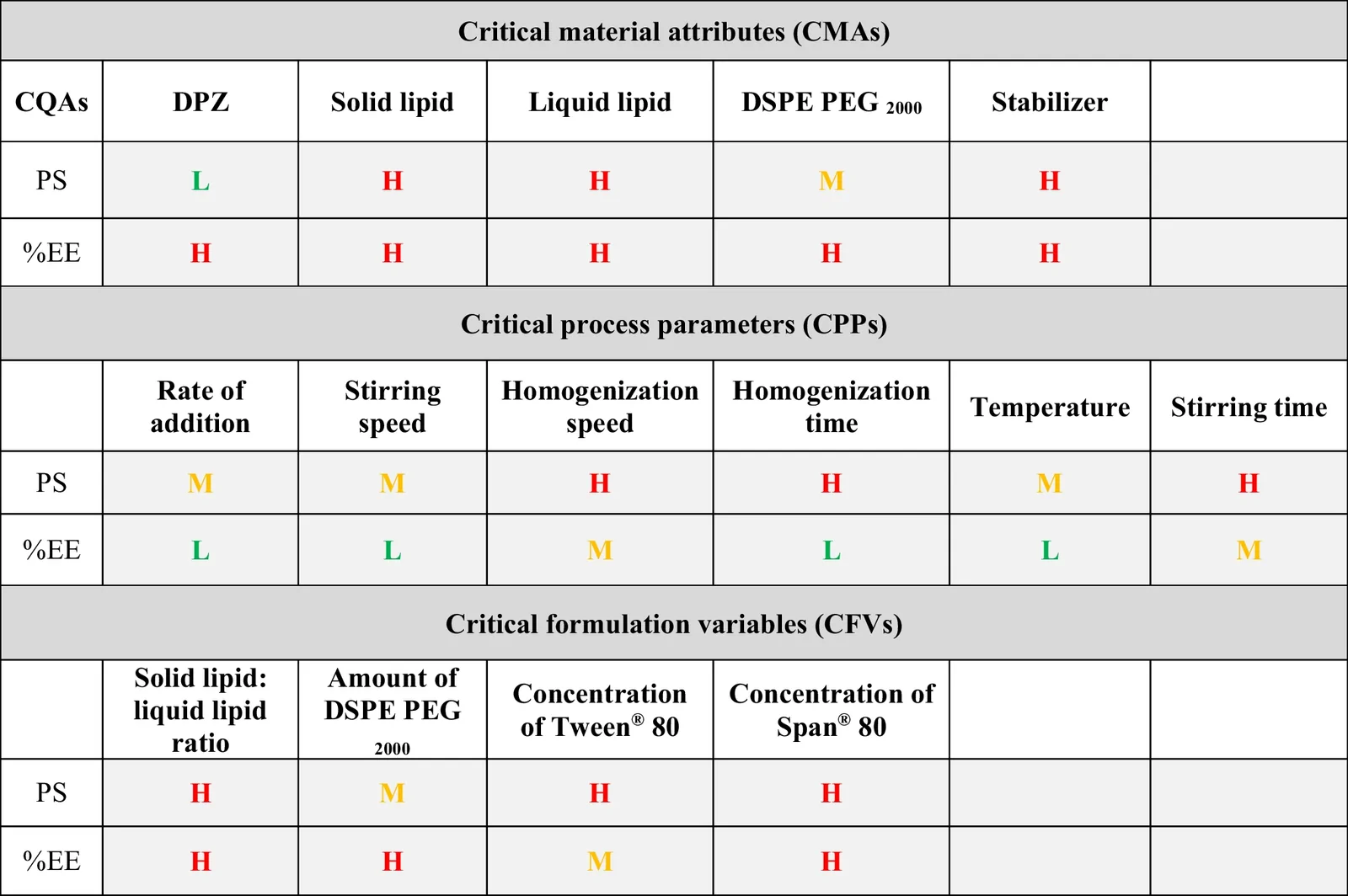
Initial[TJ1] REM-based risk assessment of DPZ-PEGylated NLCs, classifying potential risks associated with CMAs, CFVs, and CPPs (H=High, M=Medium, L=Low)

To complement the qualitative analysis, a quantitative FMEA was performed. RPN values were calculated as the product of severity, occurrence, and detectability scores for each factor (Table 3). Consistent with the REM outcomes, the solid lipid: liquid lipid ratio (RPN = 60), concentration of Tween^®^ 80 (RPN = 30), concentration of Span^®^ 80 (RPN = 45), amount of DSPE PEG_2000_ (RPN = 30), homogenization speed (RPN = 40), homogenization time (RPN = 36), and stirring time (RPN = 32) emerged as critical determinants. Parameters with RPN values ≥ 25 were classified as high risk and selected for further screening by PBD [56].

**Table 3:** FMEA analysis of DPZ-PEGylated NLCs, showing RPN scores for each factor, derived from severity, occurrence, and detectability values

Risk factor category		Occurrence (O)	Severity (S)	Detection (D)	Risk priority number (RPN = O*S*D)
Miscellaneous					
	Temperature	2	1	3	6
	Humidity	1	1	2	2
	Personal	3	1	1	3
CMAs					
Drug	Molecular weight	1	3	3	9
	logP	1	2	3	6
	Pka	1	2	2	4
	Solubility	2	2	3	12
Solid lipid & liquid lipid	Type	1	3	4	12
	Solubility	2	2	3	12
	Melting point	1	2	3	6
	Viscosity	2	2	4	16
Stabilizers	Type	1	1	5	5
	HLB	2	1	4	8
	Solubility	4	1	3	12
	Viscosity	2	1	4	8
DSPE PEG_2000_	Molecular weight	1	3	3	9
	Viscosity	2	2	4	16
Mili Q water		1	1	2	2
CFVs					
	**Solid lipid: liquid lipid ratio**	3	4	5	**60**
	**Concentration of Tween**^®^ **80**	3	2	5	**30**
	**Concentration of Span**^®^ **80**	3	3	5	**45**
	**Amount of DSPE PEG**_**2000**_	2	3	5	**30**
CPPs					
	**Homogenization speed**	2	4	5	**40**
	**Homogenization time**	3	3	4	**36**
	**Stirring time**	2	4	4	**32**
	Stirring speed	2	2	4	16
	Rate of addition	2	2	5	20
	Temperature	1	1	4	4

### Screening of significant factors by PBD

Preliminary screening studies identified solid lipid: liquid lipid ratio, concentration of Tween^®^ 80, concentration of Span^®^ 80, amount of DSPE PEG_2000_ homogenization speed, homogenization time, and stirring time as key components influencing the preparation of DPZ-PEGylated NLCs. A two-level, seven-factor PBD was employed to assess the significance of formulation and process variables (Table S3). Fifteen experimental runs, including three centre points, were generated, resulting in PEGylated NLCs with PS ranging from 98.4 ± 1.6 to 258.5 ± 2.3 nm and %EE between 78.7 ± 1.4% and 88.7 ± 1.5% (Table S4). The statistical model was reduced to retain only significant factors, while the others were pooled into the error term [57].

**Fig. 2:**
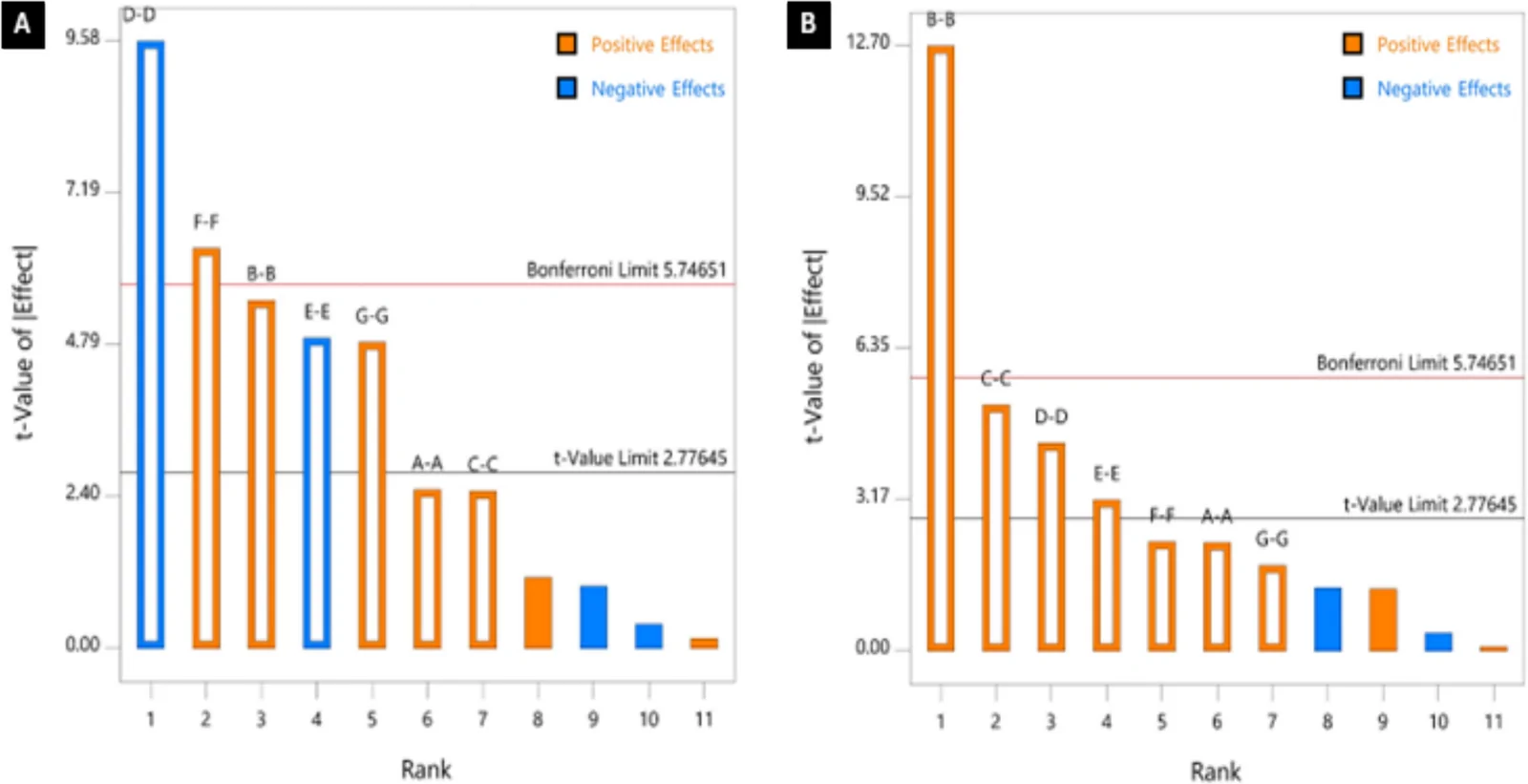
Pareto charts representing the screening of formulation and process factorsfor critical quality attributes (CQAs) using PBD: (**A**) mean PS (nm) and (**B**) EE (%). Factors are coded as follows: A-A, amount of DSPE PEG_2000_; B-B, solid lipid: liquid lipid ratio; C–C, concentration of Tween^®^ 80; D-D, concentration of Span^®^ 80; E-E, homogenization speed; F-F, homogenization time; and G-G, stirring time. Bars above the t-value limit indicate statistically significant factors, with positive and negative effects shown in orange and blue, respectively

Pareto charts (Figs. 2 A and 2B) and ANOVA revealed the factors most strongly affecting the CQAs. Rank in the screening design indicates highly significant to less significant factors (1-Highly significant,10-less significant). Factors with bars above the Bonferroni limit in the Pareto chart indicate highly significant effects on the response and are considered critical formulation variables, as their influence is statistically robust and unlikely to occur by chance. For PS, significant factors (*p* < 0.05) included solid lipid: liquid lipid ratio, concentration of Span^®^ 80, homogenization speed, homogenization time, and stirring time. For %EE, the solid lipid: liquid lipid ratio, concentration of Tween^®^ 80, concentration of Span^®^ 80, and homogenization speed were significant contributors. Amount of DSPE PEG_2000_, although relevant for mucoadhesion, did not significantly affect the selected CQAs in this screening phase. The common significant factors across both responses were the solid lipid: liquid lipid ratio, concentration of Span^®^ 80, and homogenization speed (*p* < 0.05). These were therefore selected as the independent factors for subsequent CCD-based optimization [58].

### Evaluation of DPZ-PEGylated NLCs

The screening batches of DPZ-PEGylated NLCs exhibited PS ranging from 98.4 ± 1.65 to 258.5 ± 2.38 nm, with a mean PDI of 0.234 ± 0.045, indicating a relatively narrow size distribution. The ZP values averaged − 21.12 ± 1.34 mV, suggesting sufficient surface charge for colloidal stability. %EE across the batches ranged from 78.7 ± 1.43% to 88.7 ± 1.58%, confirming efficient entrapment of DPZ within the lipid matrix.

### Systematic formulation optimization of DPZ-PEGylated NLCs

A CCD was employed to evaluate the influence of three independent factors, namely the solid lipid: liquid lipid ratio, concentration of Span^®^ 80, and homogenization speed, on the dependent responses PS (Y_1_) and %EE (Y_2_), with the coded and decoded factor levels provided in Table 4. Nineteen experimental runs were generated, comprising eight factorial points, six axial points, and five centre points. The resulting data were fitted to polynomial models (linear, quadratic, and two-factor interaction), and model adequacy was assessed using the coefficient of variation (CV), adjusted R^2^, and predicted R^2^ values. ANOVA further confirmed the statistical significance of the selected models and the contribution of individual factors to each response [59].

**Table 4:** Coded and decoded factors and their levels used in CCD

Independent factors	Units	Levels				
		**Coded value**				
		−1.414	−1	0	+ 1	+ 1.414
A-Solid lipid: liquid lipid ratio	Ratio	−1.414	−1	0	+ 1	+ 1.414
B-Concentration of Span^®^ 80	%	−1.414	−1	0	+ 1	+ 1.414
C-Homogenization speed	rpm	−1.414	−1	0	+ 1	+ 1.414
**Independent factors**	**Units**	**Levels**				
		**Decoded value**				
		−1.414	−1	0	+ 1	+ 1.414
A- Solid lipid: liquid lipid ratio	Ratio	20:80	30:70	50:50	70:30	80:20
B-Concentration of Span^®^ 80	%	0.5	1.5	2.5	3.5	4.5
C-Homogenization speed	rpm	4.2	5.2	6.2	7.2	8.2
Dependent factors	Constraints					
Y_1_- PS (nm)	Minimum < 150 nm					
Y_2_- EE (%)	Maximum > 85%					

#### *Response (Y*_*1*_*): PS*

The PS of DPZ-PEGylated NLCs generated through CCD ranged from 88.2 ± 3.7 nm to 534.5 ± 2.1 nm (Table 5). ANOVA indicated that the quadratic model best described the response, with a highly significant p-value (< 0.0001) and a nonsignificant lack-of-fit (*p* = 0.6714, F = 0.6655), confirming the model’s suitability (Table S5). Regression statistics further demonstrated robustness, with R^2^, adjusted R^2^, and predicted R^2^ values of 0.9999, 0.9999, and 0.9997, respectively, and a negligible difference (0.0002) between adjusted and predicted R^2^. The adequate precision value was 428.49, well above the required threshold of 4, confirming a high signal-to-noise ratio and reliable exploration of the design space.

**Table 5:** CCD design matrix with observed responses Y_1_ (PS) and Y_2_ (%EE) presented as (mean ± SD, n = 3), used to identify optimized conditions for DPZ-PEGylated NLCs (* = *centre point batches*)

Std run	Factor A	Factor B	Factor C	Response 1 Y_1_ PS (nm)	Response 2 Y_2_ EE (%)
	A: Solid lipid: Liquid lipid ratio (ratio)	B-Con of Span^®^ 80 (%)	C-Homo speed (rpm)		
11	0	−1.68179	0	282.2 ± 3.27	84.6 ± 2.13
16*	0	0	0	138.5 ± 2.26	90.0 ± 1.42
19*	0	0	0	135.2 ± 1.22	92.4 ± 1.36
7	−1	1	1	95.12 ± 3.45	82.7 ± 2.48
3	−1	1	−1	280.3 ± 2.31	81.5 ± 1.59
10	1.68179	0	0	165.6 ± 3.21	81.2 ± 2.13
5	−1	−1	1	168.2 ± 2.64	77.9 ± 2.78
2	1	−1	−1	402.4 ± 3.68	82.2 ± 2.13
13	0	0	−1.68179	522.2 ± 2.35	81.5 ± 2.38
1	−1	−1	−1	534.5 ± 2.14	79.1 ± 1.23
4	1	1	−1	240.1 ± 2.76	86.1 ± 2.34
15*	0	0	0	137.3 ± 2.15	91.8 ± 1.67
9	−1.68179	0	0	250.2 ± 3.26	78.6 ± 2.14
14	0	0	1.68179	116.3 ± 2.12	79.3 ± 2.35
17*	0	0	0	138.5 ± 2.19	91.0 ± 1.67
6	1	−1	1	100.4 ± 3.28	78.3 ± 2.34
8	1	1	1	125.6 ± 2.45	84.7 ± 2.13
12	0	1.68179	0	88.2 ± 3.76	88.8 ± 1.62
18*	0	0	0	139.4 ± 2.13	92.2 ± 1.72

Among the independent factors, the solid lipid: liquid lipid ratio (Factor A), concentration of Span^®^ 80 (Factor B), and homogenization speed (Factor C) were all highly significant (p < 0.0001). Model terms A, B, C, AB, AC, BC, A^2^, B^2^, and C^2^ contributed significantly to PS (*p* < 0.05), and the polynomial regression equation was derived as

6 $$Particle\; size=+137.80 -25.84\text{ A }-57.87\text{ B }-120.88\text{ C }+23.75\text{ AB }+16.75\text{ AC }+46.00\text{ BC }+24.60 {\mathrm{A}}^{2}+16.65 {\mathrm{B}}^{2}+64.02 {\mathrm{C}}^{2}$$

**Fig. 3:**
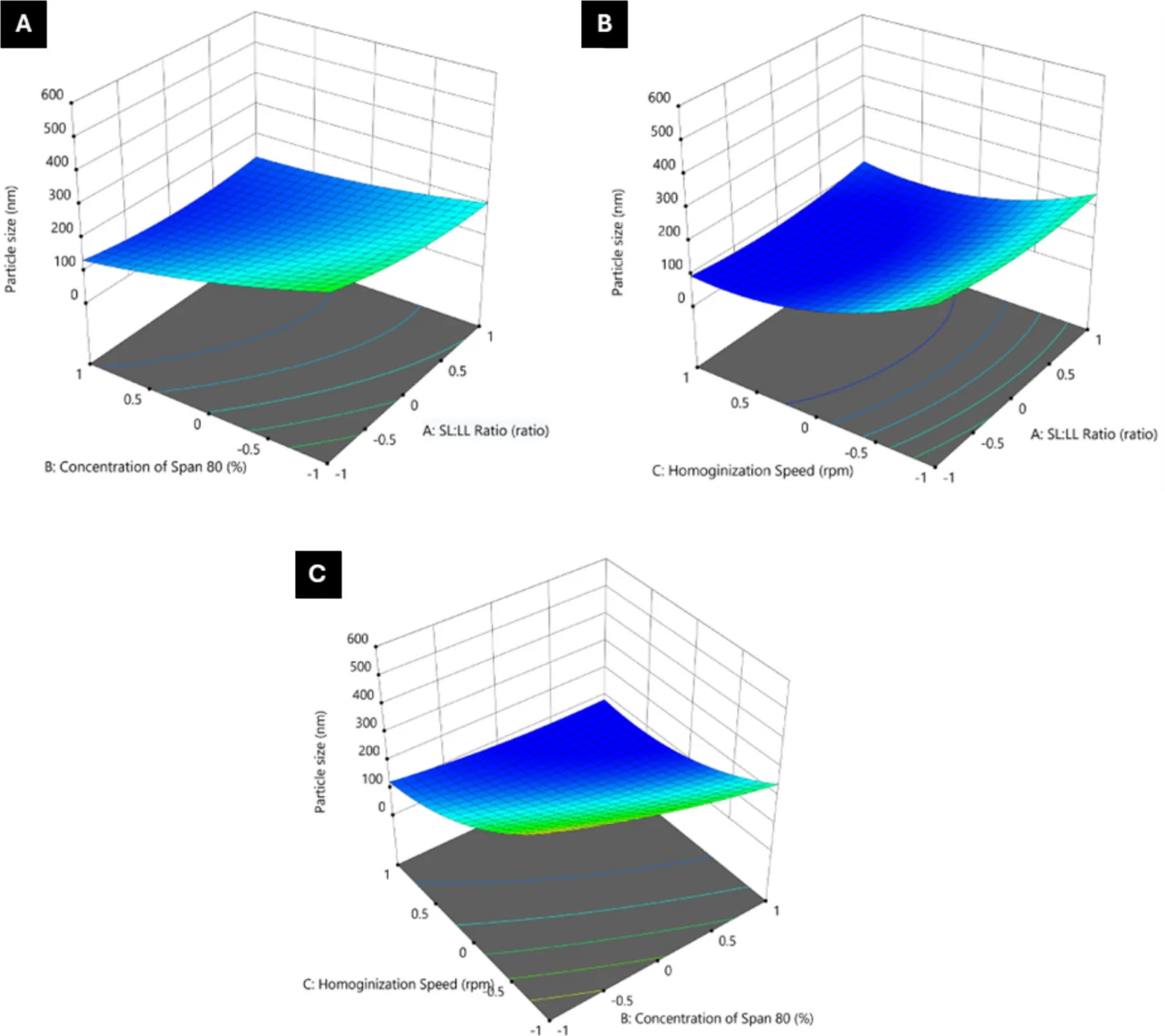
Three-dimensional response surface plots showing the effect of formulation and process factors on PS (nm) (Y_1_): (**A**) interaction between solid lipid: liquid lipid ratio and concentration of Span^®^ 80; (**B**) interaction between solid lipid: liquid lipid ratio and homogenization speed; and (**C**) interaction between concentration of Span^®^ 80 and homogenization speed

The influence of each factor is illustrated in the 3D response surface plots (Figs. 3A–C). A reduction in PS was observed as the solid lipid: liquid lipid ratio increased from − 1 to 0, followed by an increase at higher ratios. This behaviour likely reflects an optimal balance of solid and liquid lipids, where small particles form at intermediate ratios, but higher solid lipid content increases viscosity and particle growth. Similar findings were reported by Puglia et al., who noted that the incorporation of liquid lipid enhances matrix mobility and favours smaller particle formation [60]. Span^®^ 80 concentration exhibited a biphasic effect: an initial increase reduced PS, attributed to decreased interfacial tension and steric stabilization at the nanoparticle surface, whereas higher concentrations caused PS to increase, likely due to deposition of excess surfactant molecules on the NLC surface. Comparable trends have been reported in earlier studies. Homogenization speed showed an inverse relationship with PS, with higher speeds yielding smaller particles. This is consistent with previous findings by Kovacevic et al., who demonstrated that greater shear forces during homogenization reduce droplet size and promote the formation of smaller nanoparticles [61].

#### *Response (Y*_*2*_*): %EE*

The %EE of DPZ-PEGylated NLCs ranged from 77.9 ± 2.7% to 92.4 ± 1.3%. ANOVA indicated that the quadratic model best described the response, with a significant p-value (< 0.0001) and a nonsignificant lack-of-fit (*p* = 0.5887, F = 0.8283), confirming model adequacy. Regression statistics further supported reliability, with R^2^, adjusted R^2^, and predicted R^2^ values of 0.9857, 0.9714, and 0.9288, respectively, and a negligible difference (0.0426) between adjusted and predicted R^2^. The adequate precision value of 21.57, well above the threshold of 4, confirmed a high signal-to-noise ratio and the model’s ability to explore the design space effectively (Table S6). Factors A (solid lipid: liquid lipid ratio), B (concentration of Span^®^ 80), and C (homogenization speed) all showed significant effects on %EE (*p* < 0.05). Model terms A, B, C, BC, A^2^, B^2^, and C^2^ were retained as significant contributors, and the reduced polynomial equation was derived as below:

7 $${\% EE }= +91.46 +0.9186\text{ A }+1.55\text{ B }-1.14\text{ C}+0.6875\text{ AB }-0.1875\text{ AC }+0.9375\text{ BC }-4.20 {\mathrm{A}}^{2}-1.90 {\mathrm{B}}^{2}-3.85 {\mathrm{C}}^{2}$$

**Fig. 4:**
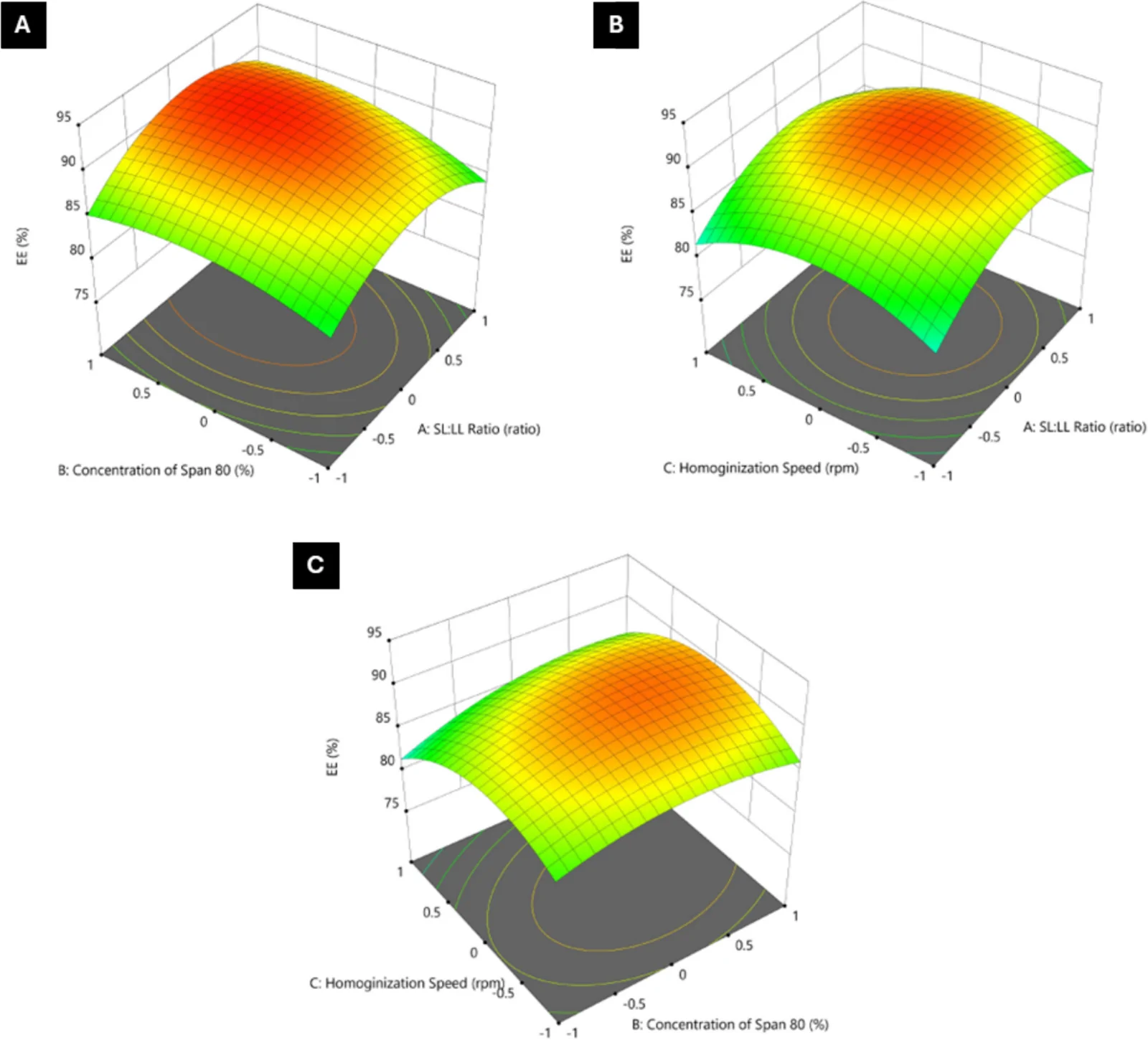
Three-dimensional response surface plots showing the effect of formulation and process factors on EE, % (Y_2_): (**A**) interaction between solid lipid: liquid lipid ratio and concentration of Span^®^ 80; (**B**) interaction between solid lipid: liquid lipid ratio and homogenization speed; and (**C**) interaction between concentration of Span^®^ 80 and homogenization speed

According to the regression model and 3D response surface plots (Figs. 4A–C), %EE was positively correlated with the solid lipid: liquid lipid ratio and concentration of Span^®^ 80 but negatively correlated with homogenization speed. An increase in solid lipid: liquid lipid ratio initially enhanced %EE due to improved solubilization of DPZ in molten Dynasan^®^ 116; however, further increases reduced liquid lipid content, limiting drug accommodation in the matrix and promoting drug expulsion. Similarly, moderate concentrations of Span^®^ 80 enhanced drug solubilization and partitioning between lipid and aqueous phases, while excessive surfactant levels saturated the matrix, destabilising drug entrapment and reducing %EE. Homogenization speed showed an inverse effect on %EE: higher shear forces produced smaller particles with larger surface area, which facilitated drug diffusion out of the lipid matrix, thereby lowering entrapment. These trends are consistent with earlier reports on lipid-based nanocarriers [62, 63].

#### Search for optimized formulation and design space

**Fig. 5:**
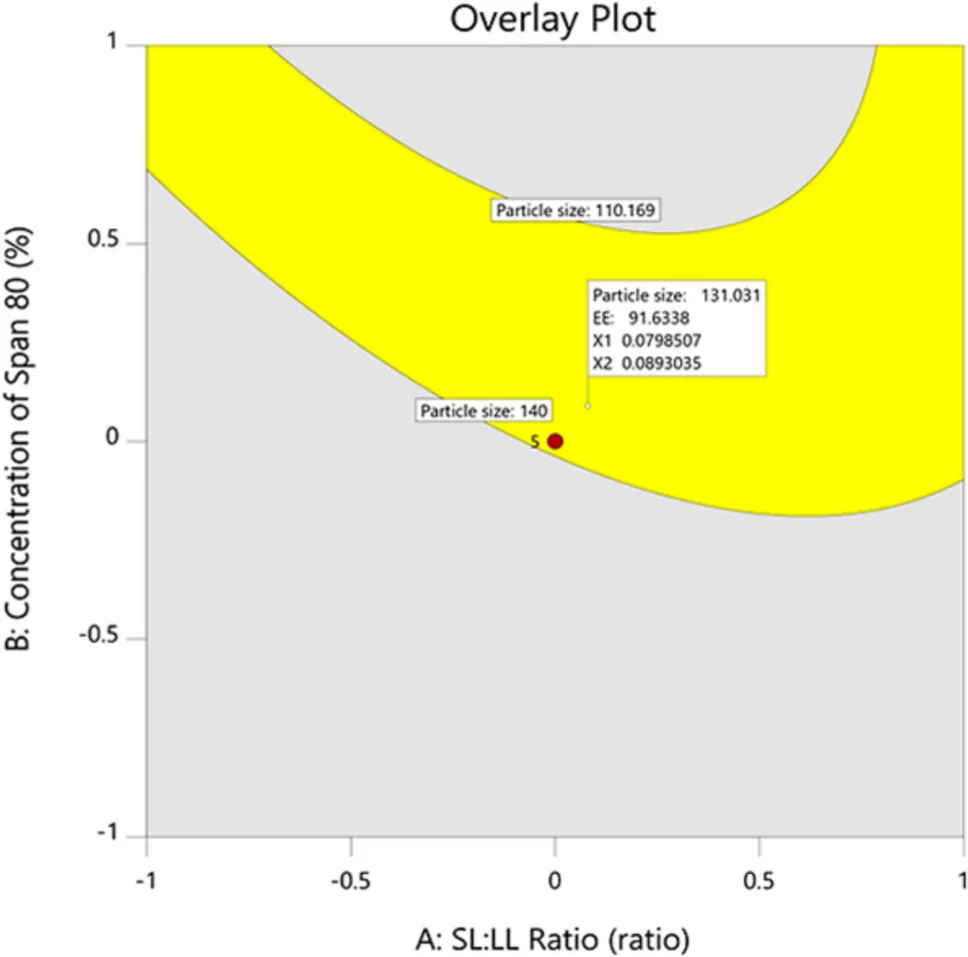
Overlay plot showing the design space for simultaneous optimization of responses Y_1_ (PS, nm) and Y_2_ (EE, %). The yellow region represents feasible conditions that meet the predefined constraints, while the grey region indicates non-feasible conditions. The selected optimal point lies within the yellow region, supporting the prediction of an optimized batch

Numerical and graphical optimization techniques were employed to identify the optimal formulation conditions after evaluating the influence of each independent factor on the responses. Target values for the selected CQAs (PS and %EE) were set, and numerical optimization generated multiple potential solutions, each with an associated desirability score ranging from 0 to 1. The solution with the highest desirability (0.99) was selected, yielding an optimized formulation composed of 10 mL Milli-Q water, 10 mg DPZ, 2.58% w/v surfactant, and a 52:48 solid lipid: liquid lipid ratio. Graphical optimization further validated these results by generating the design space (Fig. 5), where the yellow region indicated feasible combinations of formulation and process parameters that satisfied the predefined CQA targets, while the grey region represented non-feasible conditions.

#### Validation of the design

Three checkpoint formulations were prepared to validate the accuracy and robustness of the optimization model. The observed mean values for PS and %EE were 133.4 ± 2.91 nm and 89.5 ± 1.51%, respectively, compared with predicted values of 131.0 nm and 91.63%. The percentage prediction error was 1.50% for PS and 2.32% for %EE, confirming the reliability and validity of the developed model.

### Characterization of optimized DPZ-PEGylated NLCs

#### PS, Polydispersity Index (PDI), and ZP

The optimized DPZ-PEGylated NLCs exhibited a PS of 133.4 ± 2.91 nm with a PDI of 0.254 ± 0.08 (Fig. S1), which closely matched the value predicted by the statistical model. The narrow PDI confirmed a nearly monodisperse distribution and minimal particle aggregation, consistent with the ideal PDI threshold (< 0.3) reported for stable nanoparticulate drug delivery systems. The uncoated NLCs displayed a slightly larger PS of 148.0 ± 2.95 nm and a PDI of 0.228 ± 0.081 (Fig. S2). The 10–20 nm reduction observed upon PEGylation may be attributed to the presence of DSPE-PEG_2000_ chains on the nanoparticle surface, which impart a stabilising “umbrella-like” structure and enhance lamellarity and colloidal stability [64]. DSPE-PEG_2000_ chains extend outward from the NLC's surface and create brush like steric layer. This configuration lowers interfacial tension and facilitates the breakup of droplets into finer nano-sized particles. In addition, the steric hindrance generated by the outward-oriented PEG chains prevents particle–particle coalescence and suppresses aggregation. Together, these effects stabilize a highly curved interface and favour the formation of smaller, more uniform and stabilized NLCs. The amphiphilic nature of PEG lowers interfacial free energy during NLCs formation as PEG acts as a laminarity reducing agent, which limits particle growth and results in a smaller PS consistent with previous reports [12]**.** Similar findings were reported by Lakhani et al., who observed a smaller PS in PEGylated NLCs compared to their non-PEGylated counterparts (218 vs. ~ 570 nm) [65]. The optimized PEGylated formulation demonstrated a ZP of − 22.7 ± 1.35 mV (Fig. S3) at pH 6.4, which is the formulation pH, while the uncoated NLCs showed a slightly higher magnitude (− 27.7 ± 2.14 mV; Fig. S4). The observed negative ZP reflects the electrostatic stability of the colloidal system, and values close to − 20 mV have been widely reported to be sufficient for maintaining physical stability of lipid-based nanoparticles. Moreover, the negatively charged surface of the prepared NLCs is considered safe and suitable for IN administration, as reported in earlier studies [46]. The reduction in ZP following PEGylation is consistent with previous reports, where the ethylene oxide chains of DSPE-PEG_2000_ shifted the shear plane into the aqueous phase, thereby lowering the measured ZP independent of the carrier’s inherent surface charge. A ZP near − 20 mV is generally considered sufficient to confer colloidal stability, and the negative surface charge is regarded as safe and appropriate for IN administration [66].

#### %EE, %DL, and %DC

The optimized DPZ-PEGylated NLCs exhibited a %EE of 89.5 ± 1.51%, % DL of 12.0 ± 0.51%, and a DC of 97.2 ± 1.8%. In comparison, non-PEGylated NLCs showed a lower %EE (81.4 ± 1.1%) but a slightly higher DL (13.1 ± 0.3%). The higher %EE observed in PEGylated systems may be attributed to the incorporation of DSPE-PEG_2000_ into the lipid matrix, which induces crystal imperfections and provides additional voids to accommodate drug molecules during nanoparticle formation. PEGylation may also alter the hydrophobicity, stiffness, chain order, and tail spacing of the lipid membrane, thereby enhancing drug encapsulation. These results are consistent with previous reports, which demonstrated higher EE values in PEGylated nano-systems when compared to their non-PEGylated alternatives [67]. The slightly reduced DL in the PEGylated formulation can be explained by the additional mass contributed by DSPE-PEG_2000_, which increases the overall weight of the nanocarrier and reduces the proportion of drug relative to total nanoparticle mass.

#### Surface morphology by HRTEM

**Fig. 6:**
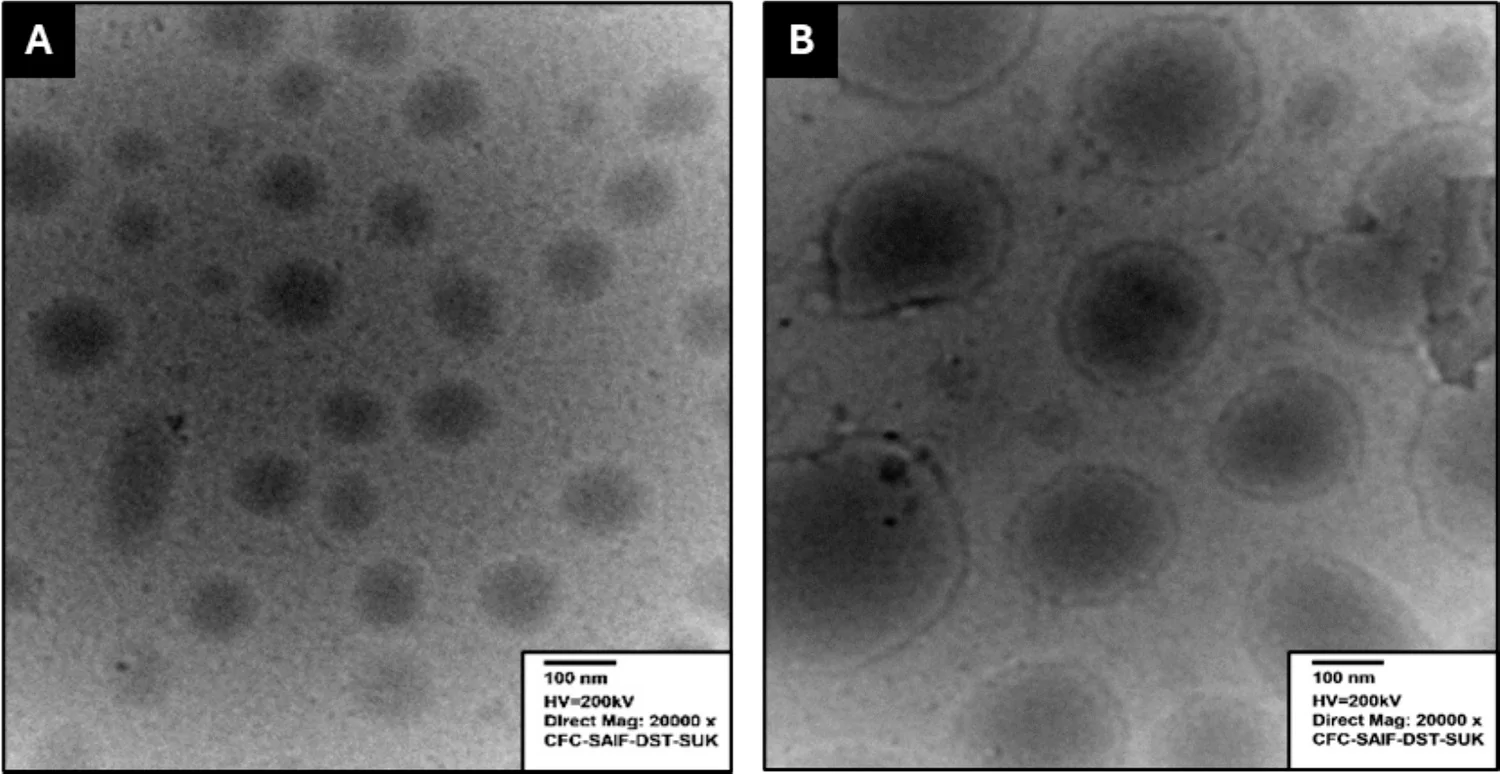
HRTEM images of (**A**) uncoated DPZ-NLCs and (**B**) optimized DPZ-PEGylated NLCs showing spherical morphology with a distinct core–shell structure

HRTEM analysis confirmed that optimized DPZ-PEGylated NLCs exhibited a well-defined spherical morphology with high uniformity in size and shape, smooth surfaces, and no visible aggregation, all indicative of good physical stability (Fig. 6 A). The optimized DPZ-PEGylated NLCs displayed an average PS of approximately 128.75 nm, which was consistent with the DLS measurement, validating agreement between microscopic and light-scattering techniques. The micrographs also revealed a distinct core–shell structure, where the dense core represented the lipid matrix encapsulating DPZ and the lighter outer shell corresponded to the DSPE-PEG_2000_ coating, confirming successful PEGylation of the nanoparticle surface. In contrast, uncoated NLCs (Fig. 6B) showed a slightly larger average PS of ~ 158.37 nm, with less distinct surface boundaries and a more irregular texture. The absence of the DSPE PEG_2000_ layer likely contributed to this increased size variation and exposed the lipid matrix directly, making the uncoated particles more prone to aggregation or fusion during storage [68].

#### Differential scanning calorimetry (DSC)

**Fig. 7:**
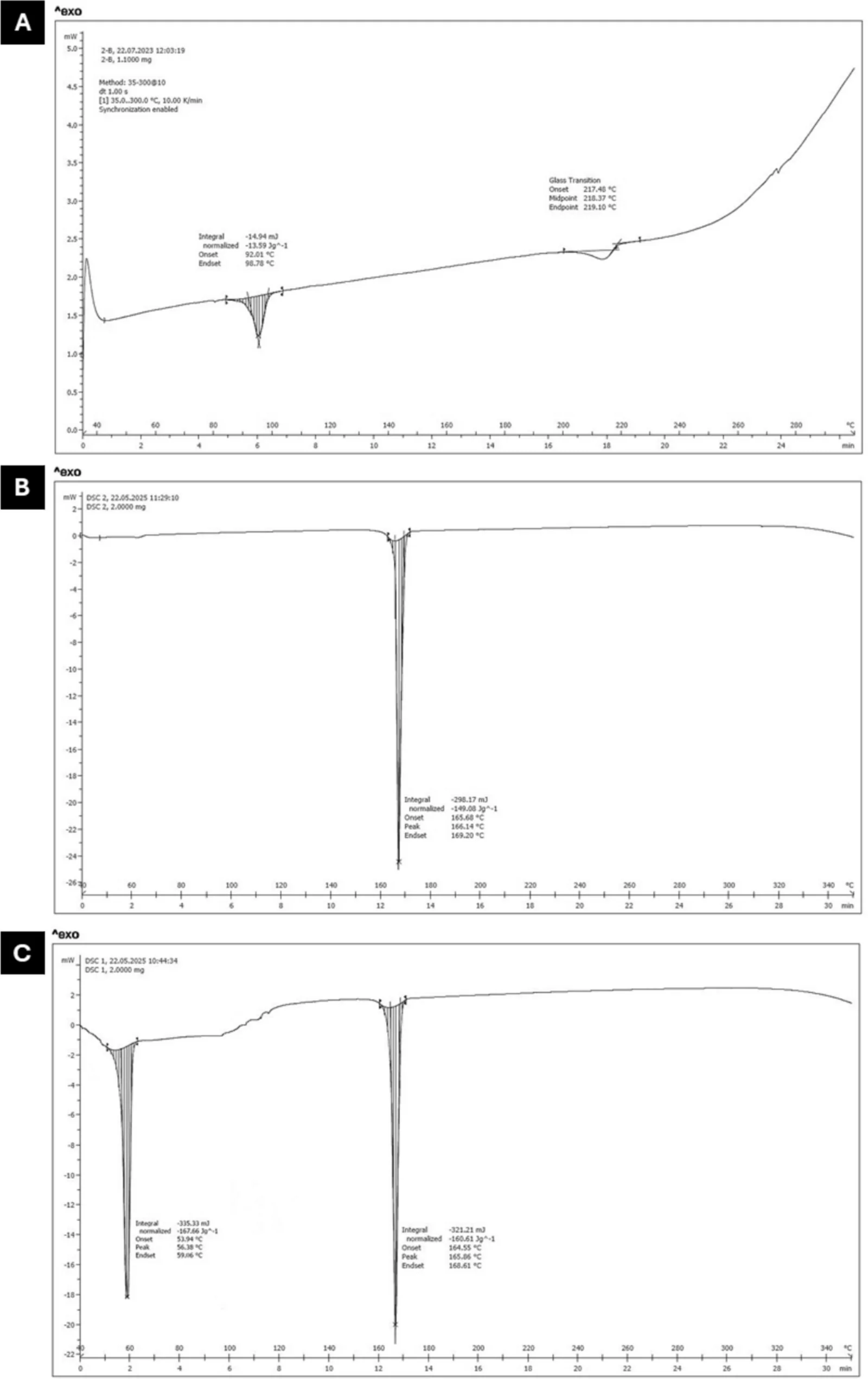
DSC thermograms of (**A**) pure DPZ, (**B**) mannitol(cryoprotectant), and (**C**) optimized lyophilized DPZ-PEGylated NLCs. The disappearance of the sharp endothermic peak of DPZ in the NLC thermogram indicates complete drug encapsulation and molecular dispersion within the lipid matrix

DSC analysis was performed to investigate the phase transition properties and crystallinity of the optimized formulation. The thermograms of pure DPZ, mannitol, and optimized lyophilized DPZ-PEGylated NLCs are shown in Fig. 7. Pure DPZ exhibited a sharp endothermic peak at 92.01 °C (Fig. 7 A), while mannitol showed a melting peak at 165.68 °C (Fig. 7B). In contrast, the thermogram of optimized lyophilized DPZ-PEGylated NLCs displayed melting transitions at 53.94 °C and 164.55 °C, corresponding to DSPE-PEG_2000_ and mannitol, respectively (Fig. 7 C). Notably, the characteristic endothermic peak of DPZ was absent in the NLC thermogram, indicating complete drug encapsulation and molecular dispersion of DPZ within the lipid matrix. This loss of crystallinity is consistent with previous reports on NLC formulations and supports improved solubility and sustained drug release [69].

#### Powder X-ray Diffraction (XRD)

**Fig. 8:**
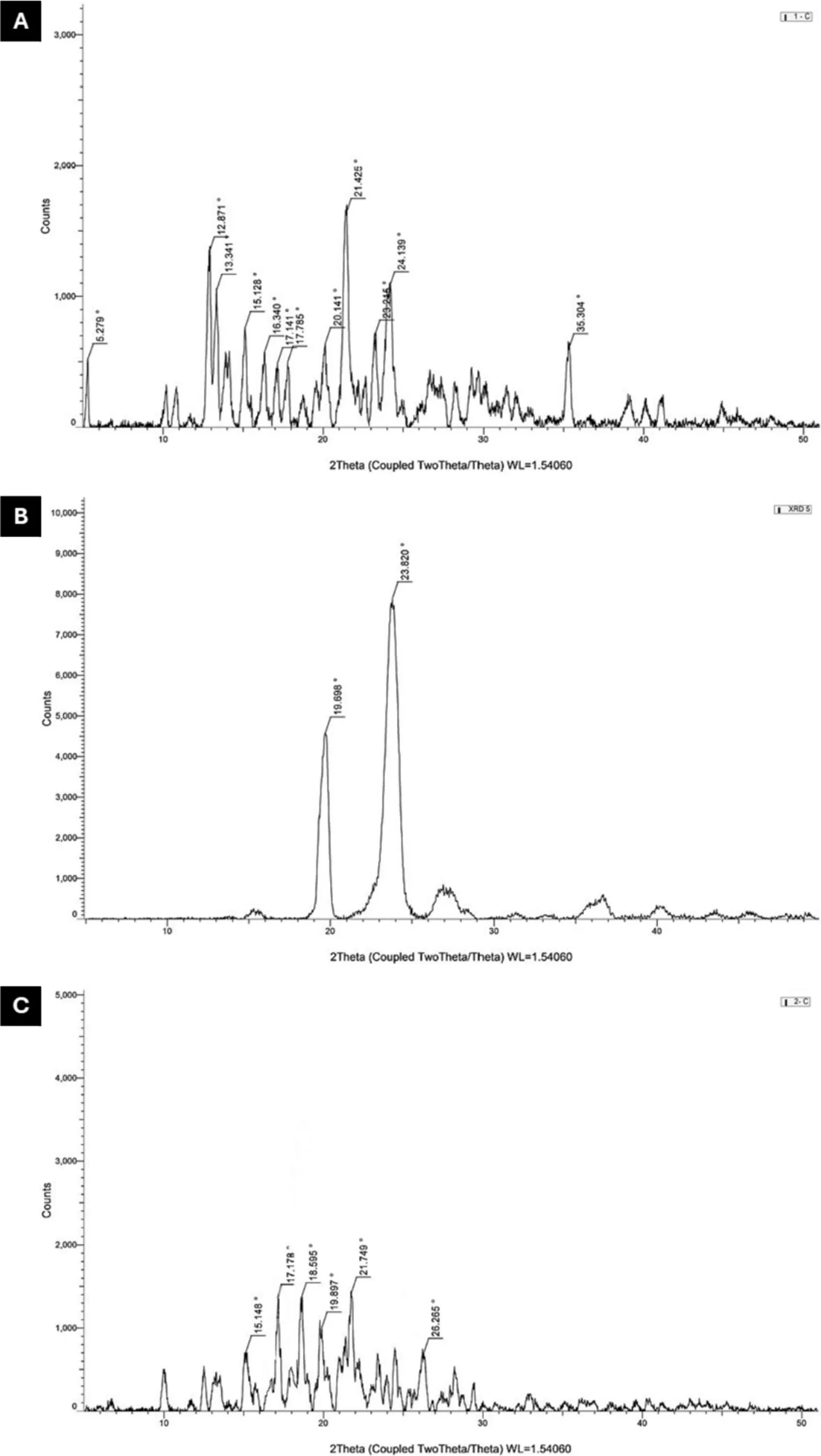
XRD patterns of (**A**) pure DPZ, (**B**) DSPE-PEG_2000_, and (**C**) optimized lyophilized DPZ-PEGylated NLCs. The disappearance of several sharp crystalline peaks of DPZ in the NLCs and the reduced overall intensity indicate loss of crystallinity and molecular dispersion of the drug within the lipid matrix, while the presence of DSPE-PEG_2000_ peaks confirms successful PEGylation

XRD was performed to evaluate the crystalline state of DPZ within the optimized formulation. Figure 8 shows the diffraction patterns of pure DPZ, DSPE-PEG_2000_, mannitol, and optimized lyophilized DPZ-PEGylated NLCs. Pure DPZ exhibited sharp and intense peaks at 5.27°, 12.87°, 13.34°, 15.12°, 16.34°, 17.14°, 17.78°, 20.14°, and 21.42° (2θ), characteristic of its crystalline nature (Fig. 8 A). DSPE-PEG_2000_ showed distinct peaks at 19.69° and 23.82° (2θ) (Fig. 8B), while mannitol presented peaks at 14.62°, 18.90°, and 23.49° (2θ). In contrast, the diffractogram of optimized lyophilized DPZ-PEGylated NLCs (Fig. 8 C) displayed reduced peak intensity and the disappearance of several DPZ-specific peaks (5.27°, 12.87°, 13.34°, 16.34°, 17.78°, and 20.14°), suggesting a marked reduction in crystallinity. The presence of peaks corresponding to mannitol and DSPE-PEG_2000_ confirmed their structural contribution, while the absence of DPZ crystalline peaks indicated that the drug was molecularly dispersed within the lipid matrix. This reduction in crystallinity corroborates the DSC findings and suggests successful encapsulation and PEGylation of the NLCs [16].

#### Mucin binding efficiency

The pH of the mucoadhesion experiment was maintained at pH 6.4, which is identical to the formulation pH. Mucin adsorption studies demonstrated a significant difference between optimized DPZ-PEGylated NLCs and uncoated DPZ-NLCs. The %MB of optimized DPZ-PEGylated NLCs was 84.6 ± 2.6%, compared to 28.6 ± 1.1% for uncoated DPZ-NLCs. PEGylation significantly enhanced mucoadhesion compared to the uncoated DPZ-NLCs. This enhancement confirms successful DSPE-PEG_2000_ surface engineering, which is expected to improve IN delivery by reducing mucociliary clearance, prolonging nasal residence time, minimizing epithelial permeation, and protecting against enzymatic degradation. The Mucoadhesion is due to the presence of DSPE–PEG_2000_ on the NLCs surface, which contributes to this through multiple mechanisms: Hydrogen bonding: The hydroxyl (–OH) groups of DSPE–PEG_2000_ can form hydrogen bonds with the glycoprotein domains of mucin. Electrostatic interactions: The negatively charged phosphate group of DSPE–PEG_2000_ can interact with positively charged amino acid residues (e.g., lysine and arginine) present in the mucus network. Polymer chain interpenetration: The flexible PEG chains are capable of interpenetrating into the mucus gel layer, promoting physical entanglement and anchoring of NLCs within the mucus [10]. These complementary mechanisms collectively support the observed mucoadhesive characteristics of the coated formulation. These results confirm the functional role of the DSPE-PEG_2000_ coating in enhancing surface interactions with mucin. Similar findings have been reported by Alavi et al. and Meng et al., who observed improved adhesion and retention of PEGylated nanoparticles in nasal tissues through in vitro and ex vivo studies [12, 48].

#### In vitro drug release and kinetic modelling

**Fig. 9:**
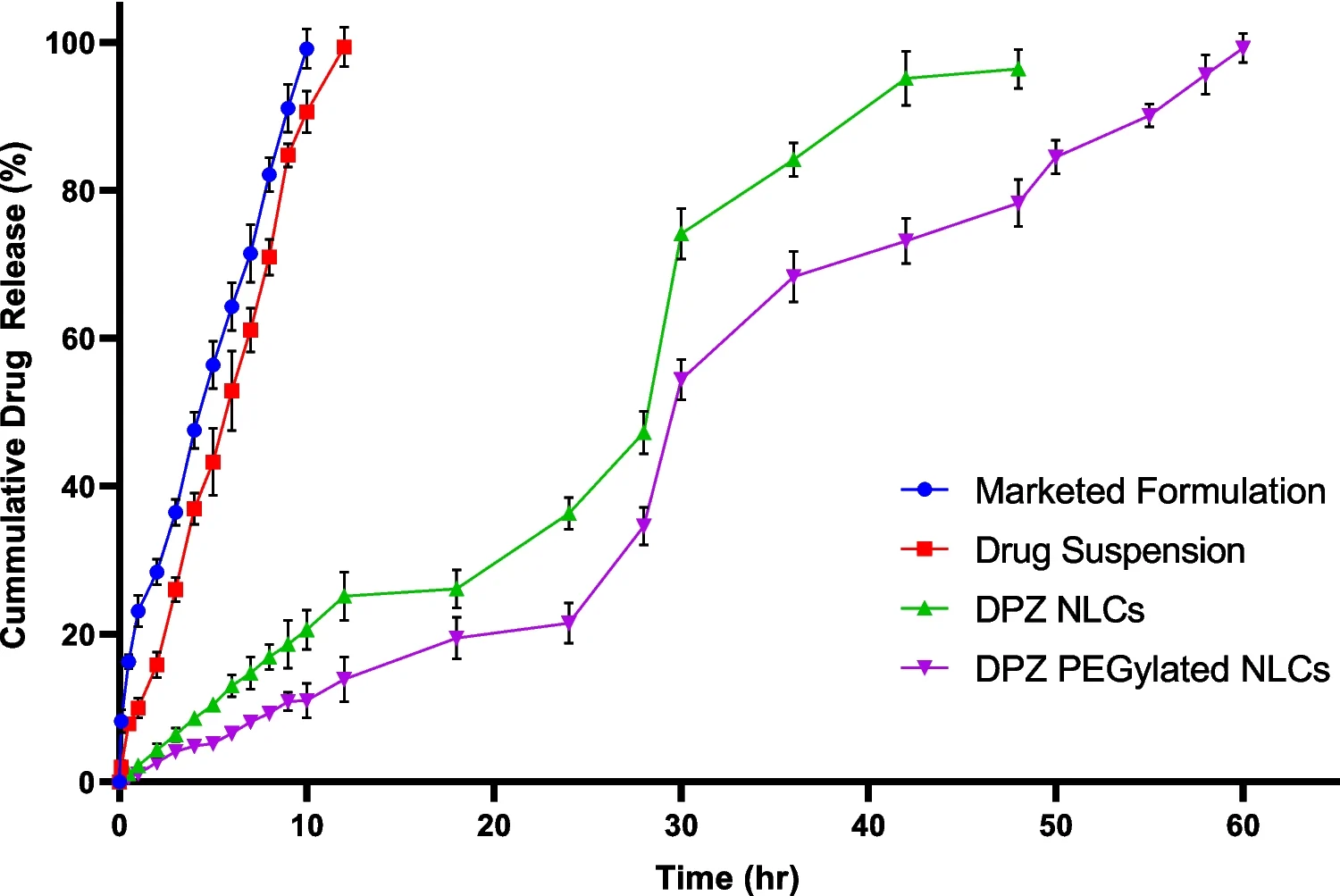
In vitro cumulative drug release profiles of DPZ suspension, marketed formulation (Donep^®^), DPZ-NLCs, and optimized DPZ-PEGylated NLCs in 0.05 M SNF (pH 6.4) at 37 ± 0.5 °C. PEGylated NLCs exhibited a slower and more sustained release compared to uncoated NLCs, suspension, and marketed formulation, confirming the role of PEGylation in prolonging drug release

The in vitro release profiles of DPZ from optimized DPZ-PEGylated and uncoated NLCs were compared against a free drug suspension and a marketed oral formulation (Donep^®^) (Fig. 9**, **Table S7). The drug suspension exhibited rapid dissolution, releasing more than 84% of DPZ within 10 h and achieving complete release by 12 h, consistent with the fast dissolution behaviour of the free drug. A similar release trend was observed for the marketed formulation, which also demonstrated near-complete release within 10 h. In contrast, DPZ-NLCs displayed a biphasic release pattern, with an initial burst of approximately 20% within the first 10 h, followed by a slower, diffusion-controlled phase that extended to 48 h, where cumulative release reached around 96%. PEGylated NLCs exhibited the most sustained and controlled release, with only 15% of the drug released at 12 h and a gradual, near-linear release extending to 60 h, at which point the cumulative release reached approximately 99%. These findings confirm that DSPE-PEG_2000_ surface modification markedly reduced drug diffusion from the lipid matrix, imparting a more controlled release compared to uncoated NLCs [70].

Kinetic modelling of the in vitro release profiles (Table S8) demonstrated distinct release behaviours across the tested formulations. For both nanocarrier systems, the Korsmeyer–Peppas model provided the best overall fit, with R^2^ = 0.9812 for DPZ NLCs and 0.9869 for PEGylated NLCs, indicating that the release process cannot be explained by simple Fickian diffusion alone. The release exponent (n) values (0.45–0.89) suggest anomalous or non-Fickian transport, where drug release is governed by the combined effects of diffusion through the lipid matrix and structural relaxation or erosion of the carrier. The zero-order model (R^2^ = 0.9784) also showed strong correlation, indicating a near-linear and sustained release pattern, particularly for PEGylated NLCs, which maintained a controlled release up to 60 h. In contrast, the drug suspension (R^2^ = 0.9941) and marketed formulation (R^2^ = 0.9902) best followed the zero-order model, consistent with their rapid, dissolution-driven release and lack of sustained delivery potential. Comparatively lower R^2^ values for the first-order, Higuchi, and Hixson–Crowell models indicate that these models only partially capture the overall release mechanism. Mechanistically, all nanocarrier systems exhibited a biphasic release pattern, characterised by an initial burst phase due to desorption of surface-associated DPZ, followed by a slower, diffusion-controlled phase. Notably, PEGylated NLCs demonstrated the most prolonged release (up to 60 h), outperforming uncoated NLCs (~ 48 h) and the suspension (< 12 h), confirming that PEGylation enhances matrix stability and diffusion control, yielding a sustained and predictable release profile. This behaviour, consisting of an initial rapid release of surface-associated DPZ followed by a sustained diffusion-controlled phase, aligns with prior reports of PEGylated NLCs providing extended release compared to their uncoated counterparts. Mahmood et al. reported a similar Korsmeyer–Peppas release profile for PEGylated acyclovir NLCs, while Zaky et al. demonstrated that PEGylated apixaban NLCs exhibited slower drug release than uncoated formulations [71]. Likewise, Wang et al. showed that PEGylated NLCs of biochanin A produced a rapid initial release followed by a markedly slower release phase [23]. These reports corroborate the present findings, confirming that PEGylation enhances sustained release characteristics of NLCs.

#### Ex vivo drug diffusion study

**Fig. 10:**
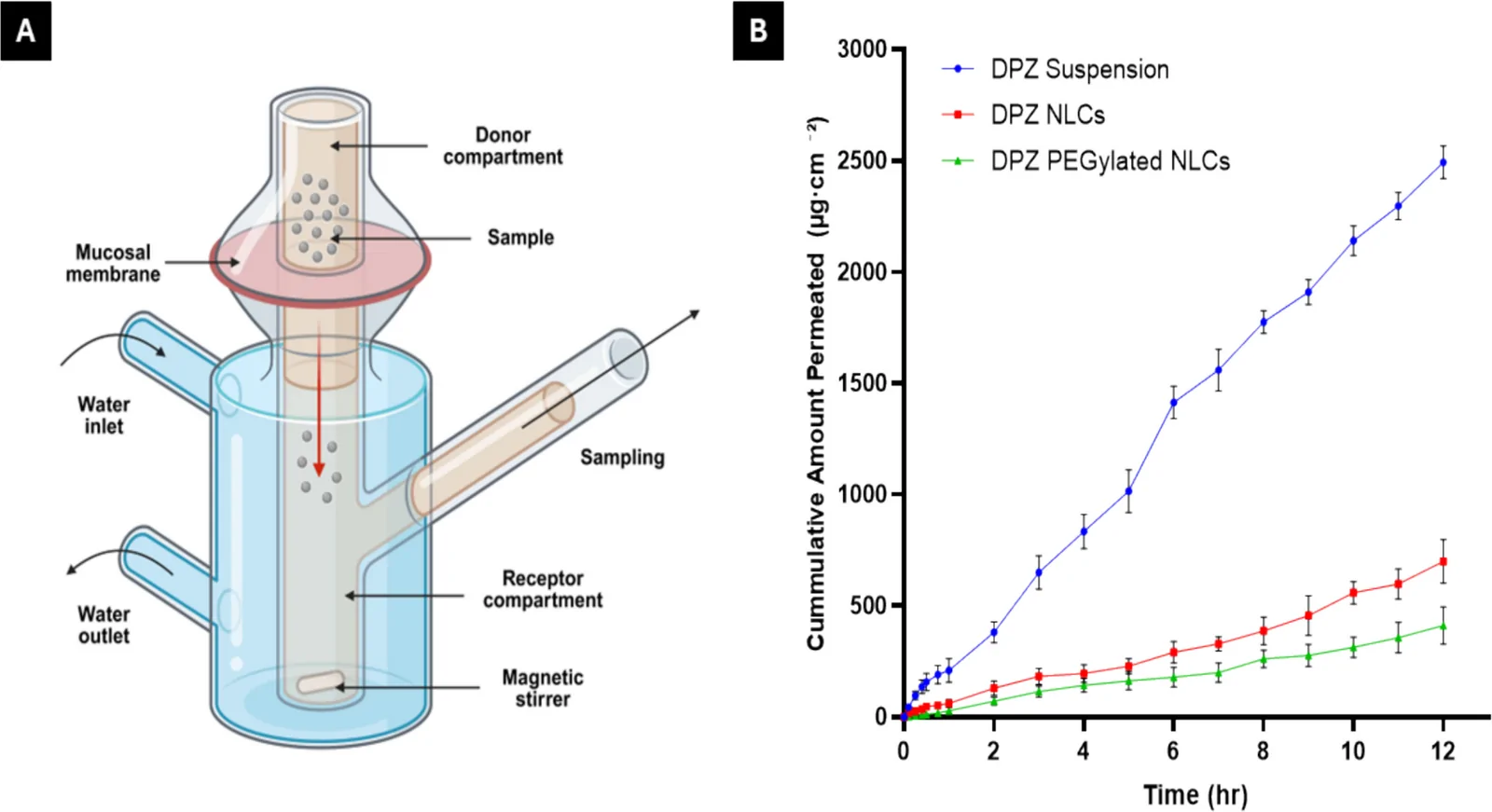
Ex vivo permeation of DPZ across goat nasal mucosa using Franz diffusion cells. (**A**) Schematic illustration of the diffusion cell setup. (**B**) Cumulative drug permeation of DPZ from DPZ suspension, DPZ-NLCs, and optimized DPZ-PEGylated NLCs in 0.05 M SNF (pH 6.4) across the nasal mucosa at 37 ± 0.5 °C. The DPZ from optimized DPZ PEGylated NLCs demonstrated the slowest and most sustained permeation compared with uncoated NLCs and the suspension, reflecting stronger interactions with mucin and the additional diffusional resistance imparted by PEGylation (*n* = 3)

Ex vivo permeation studies were performed using freshly excised goat nasal mucosa mounted on vertical Franz diffusion cells, with the epithelial side facing the donor compartment (Fig. 10 A). The cumulative permeation profiles of DPZ from suspension, NLCs, and optimized PEGylated NLCs are shown in Fig. 10B**,** and the corresponding quantitative parameters are summarized in Table S9. Consistent with its rapid dissolution in vitro, the DPZ from DPZ suspension showed fast diffusion across the mucosa, with approximately 84.7% permeation at 12 h. By contrast, DPZ from NLCs exhibited a markedly attenuated profile with only ~ 23% release at 12 h, while DPZ from PEGylated NLCs showed the most prolonged diffusion, reaching just ~ 12.1% at the same time point.

The permeability of DPZ from PEGylated NLCs observed within the 12 h window appears lower than that from DPZ suspension and non-PEGylated NLCs. The permeability experiment was limited to 12 h due to tissue-viability constraints, beyond which structural integrity and physiological relevance of the tissue could not be assured. However, an extended-duration study was subsequently performed under acceptable conditions, and the results demonstrated that approximately 93.24 ± 3.29% of the DPZ permeated by 67 h from optimized PEGylated NLCs Table S9, confirming that higher amounts of DPZ continue to diffuse over time and eventually reach the acceptor compartment. These findings collectively indicate that PEGylated NLCs are capable of delivering DPZ at an adequate level for CNS uptake, while supporting a more sustained permeation profile [72, 73].

This reduced permeation from nanoparticle-based formulations reflects a more controlled diffusion process, an advantageous characteristic for nose-to-brain delivery, where prolonged mucosal retention and sustained drug release are desired. To quantify the diffusion kinetics, steady-state flux (J _ss_) and apparent permeability coefficients (P_app_) were calculated from the linear portion of the permeation curves. The results are summarized in Table 6.

**Table 6:** Ex vivo permeation parameters of DPZ from different formulations across goat nasal mucosa

Formulations	J_ss_ (µg/cm^2^·h)	P_app_ (cm/h)	P_app_ (cm/s)
DPZ Suspension	181.453	0.181453	5.04 × 10⁻^5^
DPZ NLCs	59.571	0.059571	1.66 × 10⁻^5^
DPZ PEGylated NLCs	43.095	0.043095	1.20 × 10⁻^5^

Quantitative analysis of the permeation kinetics further confirmed these trends. The DPZ from DPZ suspension displayed the highest steady-state flux (J_ss_ = 181.453 µg/cm^2^·h) and apparent permeability coefficient (P_app_ = 0.181453 cm/h; 5.04 × 10⁻^5^ cm/s), reflecting rapid passive diffusion of the drug. By comparison, DPZ from DPZ NLCs demonstrated a significantly lower flux (J_ss_ = 59.571 µg/cm^2^·h) and permeability (P_app_ = 0.059571 cm/h; 1.66 × 10⁻^5^ cm/s), while DPZ from optimized PEGylated NLCs exhibited the lowest flux (J_ss_ = 43.095 µg/cm^2^·h) and permeability (P_app_ = 0.043095 cm/h; 1.20 × 10⁻^5^ cm/s). This order: Suspension > NLC > PEG-NLC, highlights the shift from rapid, uncontrolled permeation to a more regulated diffusion profile as the formulation complexity increases. Such a pattern is commonly reported for nanoparticle-based nasal delivery systems and is mechanistically logical: the free drug in suspension has no diffusional barriers, establishing the steepest concentration gradient and enabling immediate passive transport. In contrast, encapsulation within a lipid matrix introduces a release-dependent permeation step that inherently slows the process [24]. The lower flux observed of DPZ from DPZ NLCs compared to the DPZ suspension can be attributed to the encapsulation of DPZ within the lipid matrix, which reduces the immediate availability of free drug and necessitates its release before permeation. Additionally, the lipid excipients facilitate drug partitioning into the mucosal membrane and may transiently modulate membrane fluidity, contributing to improved interaction with epithelial surfaces. PEGylation further altered permeation behaviour: while PEG chains are known to enhance mucus penetration by reducing steric hindrance, their hydrophilic corona also decreases direct nanoparticle–epithelium interactions. This hydrophilic shielding can act as a steric barrier, limiting intimate contact with the epithelial surface and thereby lowering initial drug partitioning into the mucosa [74]. Moreover, reduced mucoadhesion may lead to shorter residence time at the epithelial interface, which further attenuates the permeation rate despite potentially improved mucus traversal [75]. This likely explains the reduced flux of DPZ from optimized DPZ-PEGylated NLCs compared to conventional NLCs, as reduced surface contact and limited residence time can offset the benefits of improved mucus diffusion. Importantly, while this results in lower initial flux, the trade-off is therapeutically advantageous, as the more sustained and prolonged diffusion profile supports continuous delivery over extended periods, an essential criterion for effective nose-to-brain drug transport [76].

The observed permeation behavior indicates that lipid-based formulation and PEGylation not only modulate drug transport kinetics but also impart advantageous properties for IN therapeutics.The lower flux and permeability values of DPZ from DPZ NLCs and optimized PEGylated NLCs are indicative of sustained diffusion, extended mucosal contact, and controlled release, which can collectively enhance therapeutic outcomes by improving drug bioavailability in the brain over an extended period of time. Moreover, reduced permeation rates may also mitigate systemic absorption and associated side effects, supporting the suitability of such nano-formulations for targeted CNS delivery [77].

#### Storage stability of optimized liquid DPZ-PEGylated NLCs

The physical stability of optimized liquid DPZ-PEGylated NLCs was assessed over one month under ambient (25 ± 2 °C) and refrigerated (5 ± 3 °C) conditions (Table 7). Samples were analysed at 0, 7, 14, 21, and 28 days for changes in PS and %EE. No visible colour change, gelation, or particle aggregation was observed during the study period. At refrigerated storage, PS increased only marginally from 133.2 ± 2.9 nm to 141.6 ± 3.8 nm, while %EE decreased from 89.5 ± 1.5% to 82.1 ± 3.7%. In contrast, at ambient temperature, PS increased more substantially from 133.2 ± 2.9 nm to 152.7 ± 5.7 nm, and %EE decreased from 89.5 ± 1.5% to 77.5 ± 4.8%. The greater instability observed at higher temperatures may be attributed to increased particle collisions and reduced microviscosity, promoting aggregation, as well as possible lipid phase separation that reduced drug entrapment. Overall, the results indicate that refrigerated storage conditions better preserve both PS and %EE, thereby maintaining formulation stability. These findings are consistent with earlier reports, such as Khan et al. on lopinavir-loaded NLCs and Shehata et al. on astaxanthin-loaded NLCs, both of which demonstrated superior stability of NLCs under refrigerated conditions compared to room temperature [78, 79].

**Table 7:** Stability profile of optimized liquid DPZ-PEGylated NLCs stored under refrigerated (5 ± 3 °C) and room temperature (25 ± 2 °C) conditions over 28 days, evaluated in terms of PS (nm) and EE (%). Data are presented as (mean ± SD,n = 3)

Storage time (days)	Refrigerated (5 ± 3 °C)		Room temperature (25 ± 2 °C)	
	PS (nm)	%EE	PS (nm)	%EE
0	133.2 ± 2.9	89.5 ± 1.5	133.2 ± 2.9	89.5 ± 1.5
7	135.1 ± 2.3	87.6 ± 2.3	137.8 ± 2.3	85.7 ± 2.6
14	137.7 ± 2.6	85.3 ± 1.8	142.5 ± 3.2	82.6 ± 2.5
21	139.3 ± 3.5	84.5 ± 2.8	149.6 ± 3.8	79.5 ± 3.2
28	141.6 ± 3.8	82.1 ± 3.7	152.7 ± 5.7	77.5 ± 4.8

## Conclusion

This study demonstrates the successful application of a comprehensive QbD framework for the development of PEGylated NLCs encapsulating DPZ for nose-to-brain delivery. Defining the QTPP and identifying PS and %EE as key CQAs ensured that formulation development was systematically aligned with the desired safety, efficacy, and performance profiles. Structured risk assessment using Ishikawa diagramming, REM, and FMEA, followed by PBD screening and CCD optimization, enabled precise identification of critical factors, establishment of a robust design space, and enhanced formulation predictability.

The optimized DPZ-PEGylated NLCs exhibited favourable physicochemical attributes, including nanoscale size, narrow PDI, high drug entrapment, and excellent content uniformity. PEGylation imparted a distinctive core–shell morphology, significantly enhanced mucin binding and hydration, and modulated drug release kinetics. In vitro and ex vivo evaluations revealed sustained, biphasic drug release over 60 h, controlled permeation across the nasal mucosa, and prolonged mucosal residence, all of which are essential for efficient direct brain delivery. Although PEGylation slightly reduced the steady-state flux relative to uncoated NLCs, the resulting controlled permeation and improved retention profile are advantageous for maintaining a consistent drug gradient across the nasal epithelium. Release kinetics conformed to the Korsmeyer–Peppas model, suggesting a non-Fickian transport mechanism governed by both diffusion and matrix erosion. Short-term stability studies further confirmed that refrigerated storage conditions preserve the physicochemical integrity of the formulation.

Comparison with recent studies underlines the novelty of our approach. Yasir et al. reported chitosan-coated DPZ-NLCs optimized by Box-Behnken design [80], while Tekade et al. investigated Nigella sativa oil-based NLCs using a factorial design [81]. Both studies demonstrated promising outcomes but relied on empirical or single-factor approaches and lacked comprehensive risk-based QbD optimization. The present work distinguishes itself by integrating QbD tools at every stage, from risk assessment to validation, and by introducing PEGylation as a versatile modification that enhances mucin interaction, reduces mucociliary clearance, and confers stability without relying on polymeric coatings or oil systems. Furthermore, unlike earlier work, our study combines in vitro*, *ex vivo, and stability assessments, thereby providing a more holistic preclinical evaluation.

Beyond the specific case of DPZ, this research establishes a generalizable and scalable framework for rationally engineering IN nanocarriers aimed at CNS targeting. The demonstrated balance of sustained release, mucosal residence, and mucus interaction provides a strategic foundation for overcoming challenges such as enzymatic degradation, rapid clearance, and poor BBB penetration. Future investigations should extend this work to in vivo pharmacokinetic, biodistribution, and pharmacodynamic studies, evaluate long-term safety and immunocompatibility, and explore targeting ligands or alternative PEG-lipid conjugates to further refine delivery specificity. Ultimately, this QbD-driven, PEGylated NLC platform represents a versatile and translationally relevant approach with significant implications for the next generation of IN nanomedicines for NDs.

## Supplementary Information

Below is the link to the electronic supplementary material.

ESM 1 (DOCX 468 KB)

ESM 2 (DOCX 24 KB)

## Data Availability

The data supporting the findings of this study are available upon reasonable request from the corresponding author.

## Abbreviations

AD: Alzheimer's disease
BBB: Blood–brain barrier
CNS: Central nervous system
ND: Neurological disorder
IN: Intranasal
CCD: Central composite design
CFVs: Critical formulation variables
CMAs: Critical materials attributes
CPPs: Critical process parameters
CQA: Critical quality attributes
QbD: Quality by design
DoE: Design of experiments
%EE: Entrapment efficiency
IN: Intranasal
NLCs: Nanostructured lipid carriers
ND: Neurological disorders
PS: Particle size
PBD: Plackett–Burman screening design
QTPPS: Quality target product profile
ZP: Zeta potential
