# Supplementary material for: Quality by design (QbD)-guided development of PEGylated nanostructured lipid carriers of donepezil for intranasal delivery
Source: Drug Deliv Transl Res. 2026 Feb 24;16(10):3677–701. doi: 10.1007/s13346-026-02061-5 (PMC13619771; doi:10.1007/s13346-026-02061-5)
Supplement: Supplementary file 1 [file 13346_2026_2061_MOESM1_ESM.docx]

**Supplementary tables:**

**Table S1 Selection of different parameters for the preparation of PEGylated NLC by OFAT (mean ± SD, n = 3).**

| **Parameters** | | **Particle size± S. D** | **%EE ± S. D** |
| --- | --- | --- | --- |
| Solid lipid: Liquid lipid ratio | 30:70 | 148.5±1.44 | 90.5±1.13 |
|  | 50:50 | 173.2±3.95 | 82.9±2.70 |
|  | 70:30 | 241.3±3.57 | 80.2±2.26 |
| Concentration of Tween^®^ 80 (%) | 0.75 | 151.2±2.98 | 88.2±1.23 |
|  | 1 | 139.5±3.40 | 91.6±1.06 |
|  | 1.25 | 246.3±3.78 | 86.1±1.23 |
| Concentration of Span^®^ 80(%) | 1.5 | 150.8±3.41 | 87.9±1.04 |
|  | 2.5 | 139.7±2.53 | 90.8±2.34 |
|  | 3.5 | 244.4±5.57 | 88.2±1.79 |
| Homogenization speed(rpm) | 5.2 | 153.9±3.45 | 86.2±1.42 |
|  | 6.2 | 139.4±2.36 | 91.5±1.09 |
|  | 7.2 | 228.4±3.19 | 82.6±2.34 |
| Homogenization time(min) | 10 | 178.2±4.27 | 86.2±1.79 |
|  | 15 | 138.4±2.89 | 91.5±1.50 |
|  | 20 | 259.3±3.12 | 84.2±2.16 |
| Stirring time(min) | 10 | 169.4±4.97 | 85.3±2.59 |
|  | 15 | 136.5±3.45 | 90.8±2.13 |
|  | 20 | 256.3±3.51 | 81.4±3.57 |
| Amount of DSPE PEG_2000_ (mg) | 50 | 144.7±3.56 | 83.5±2.56 |
|  | 100 | 137.2±2.67 | 90.5±2.10 |
|  | 150 | 240.5±3.42 | 81.4±2.13 |

**Table S2 The stability of optimized lyophilized DPZ-PEGylated formulation stored at 5 ±3 °C, 25 ±2°C (mean ± SD, n = 3).**

| **Storage time (days)** | **Refrigerated (5 ± 3 °C)** | | **Room temperature (25 ± 2 °C)** | |
| --- | --- | --- | --- | --- |
|  | PS (nm) | %EE | PS (nm) | %EE |
| 0 | 132.2 ± 1.3 | 88.1 ± 1.2 | 132.2 ± 1.3 | 88.1 ± 1.2 |
| 3 | 133.8 ± 1.4 | 87.5 ± 1.7 | 138.2 ± 1.9 | 87.2 ± 1.7 |
| 6 | 134.5 ± 1.7 | 87.2 ± 2.1 | 142.4 ± 2.1 | 85.5 ± 2.1 |
| 9 | 135.2 ± 2.1 | 86.7 ± 2.5 | 144.1 ± 1.6 | 84.1 ± 2.4 |
| 12 | 135.9 ± 1.2 | 85.8 ± 1.4 | 146.2 ± 1.7 | 83.6 ± 1.6 |
| 15 | 136.1 ± 1.9 | 85.1 ± 1.3 | 147.7 ± 1.5 | 82.4 ± 2.3 |

**Table S3: Coded and decoded factors with their levels used in the PBD for screening critical formulation and process variables in the development of DPZ-PEGylated NLCs.**

| **Factors** | **Factor code** | **Factors** | **Unit** | **Coded value** | |
| --- | --- | --- | --- | --- | --- |
|  |  |  |  | **Lower level (-1)** | **Higher level (+1)** |
| Factor 1 | A | Amount of DSPE PEG_2000_ | mg | -1 | +1 |
| Factor 2 | B | Solid lipid: Liquid lipid ratio | ratio | -1 | +1 |
| Factor 3 | C | Concentration of Tween^®^ 80 | % | -1 | +1 |
| Factor 4 | D | Concentration of Span^®^ 80 | % | -1 | +1 |
| Factor 5 | E | Homogenization speed | rpm | -1 | +1 |
| Factor 6 | F | Homogenization time | min | -1 | +1 |
| Factor 7 | G | Stirring time | min | -1 | +1 |
|  |  |  |  |  |  |
| **Factors** | **Factor code** | **Factors** | **Unit** | **Decoded value** | |
|  |  |  |  | **Lower level (-1)** | **Higher level (+1)** |
| Factor 1 | A | Amount of DSPE PEG_2000_ | mg | 50 | 150 |
| Factor 2 | B | Solid lipid: Liquid lipid ratio | ratio | 30:70 | 70:30 |
| Factor 3 | C | Concentration of Tween^®^ 80 | % | 0.75 | 1.25 |
| Factor 4 | D | Concentration of Span^®^ 80 | % | 1.5 | 3.5 |
| Factor 5 | E | Homogenization speed | rpm | 5.2*1000 | 7.2*1000 |
| Factor 6 | F | Homogenization time | min | 10 | 20 |
| Factor 7 | G | Stirring time | min | 10 | 20 |
| **Dependent factors** | | | | | |
| Y_1_=PS (nm) | | | | | |
| Y_2_=EE (%) | | | | | |

**Table S4: PBD matrix with coded factors and the corresponding observed responses Y_1_ (particle size, PS, nm) and Y_2_ (EE, %) for DPZ-PEGylated NLCs (mean ± SD, n = 3).**

| Std run | Factor 1 | Factor 2 | Factor 3 | Factor 4 | Factor 5 | Factor 6 | Factor 7 | Response Y_1_  PS (nm) | ResponseY_2_  EE (%) |
| --- | --- | --- | --- | --- | --- | --- | --- | --- | --- |
| 14* | 0 | 0 | 0 | 0 | 0 | 0 | 0 | 142.4±2.14 | 88.16±1.87 |
| 4 | -1 | 1 | -1 | 1 | 1 | -1 | 1 | 123.2±1.32 | 87.6±1.22 |
| 9 | 1 | 1 | 1 | -1 | -1 | -1 | 1 | 246.3±2.45 | 87.5±1.01 |
| 3 | 1 | -1 | 1 | 1 | -1 | 1 | 1 | 193.3±2.40 | 84.6±1.34 |
| 1 | 1 | 1 | -1 | 1 | 1 | 1 | -1 | 140.3±1.56 | 87.8±1.38 |
| 10 | -1 | 1 | 1 | 1 | -1 | -1 | -1 | 142.1±1.59 | 86.7±1.32 |
| 2 | -1 | 1 | 1 | -1 | 1 | 1 | 1 | 255.5±2.20 | 88.1±1.56 |
| 12 | -1 | -1 | -1 | -1 | -1 | -1 | -1 | 148.8±1.52 | 78.7±1.43 |
| 6 | -1 | -1 | -1 | 1 | -1 | 1 | 1 | 164.9±3.41 | 82.3±1.79 |
| 8 | 1 | 1 | -1 | -1 | -1 | 1 | -1 | 258.5±2.38 | 85.8±1.31 |
| 15* | 0 | 0 | 0 | 0 | 0 | 0 | 0 | 141.8±1.61 | 87.7±1.22 |
| 7 | 1 | -1 | -1 | -1 | 1 | -1 | 1 | 169.3±1.42 | 81.1±1.52 |
| 5 | -1 | -1 | 1 | -1 | 1 | 1 | -1 | 170.0±2.17 | 83.1±1.61 |
| 11 | 1 | -1 | 1 | 1 | 1 | -1 | -1 | 98.40±1.65 | 85.0±1.89 |
| 13* | 0 | 0 | 0 | 0 | 0 | 0 | 0 | 140.6±2.38 | 88.7±1.58 |

(*=centre point batches)

**Table S5: ANOVA results for response Y_1_ (particle size, PS, nm) based on the quadratic model used in CCD optimization.**

| **Source** | **Sum of square** | **df** | **Mean square** | **F-Value** | **P-Value** |
| --- | --- | --- | --- | --- | --- |
| Model | 3.377 x 10^5^ | 9 | 37517.39 | 18177.66 | <0.0001 |
| A-Solid lipid: liquid lipid ratio | 9121.82 | 1 | 9121.82 | 4419.64 | <0.0001 |
| B-Concentration of Span^®^ 80 | 45729.65 | 1 | 45729.65 | 22156.60 | <0.0001 |
| C-Homogenisation speed | 1995 x 10^5^ | 1 | 1995E+05 | 96682.45 | <0.0001 |
| AB | 4512.50 | 1 | 4512.50 | 2186.36 | <0.0001 |
| AC | 2244.50 | 1 | 2244.50 | 1087.49 | <0.0001 |
| BC | 16928.00 | 1 | 16928.00 | 8201.83 | <0.0001 |
| A^2^ | 8262.32 | 1 | 8262.32 | 4003.20 | <0.0001 |
| B^2^ | 3783.08 | 1 | 3783.08 | 1832.95 | <0.0001 |
| C^2^ | 55952.89 | 1 | 55952.89 | 27109.89 | <0.0001 |
| Residual | 18.58 | 9 | 2.06 | - | - |
| Lack of fit | 8.44 | 5 | 1.69 | 0.6655 | 0.6714 |
| Pure error | 10.14 | 4 | 2.54 | - |  |

**Table S6: ANOVA analysis of response Y_2_ EE (%) for quadratic model**

| **Source** | **Sum of square** | **df** | **Mean square** | **F-Value** | **P-Value** |
| --- | --- | --- | --- | --- | --- |
| Model | 470.55 | 9 | 52.28 | 68.96 | <0.0001 |
| A-Solid lipid: liquid lipid ratio | 11.52 | 1 | 11.52 | 15.20 | 0.0036 |
| B-Concentration of Span^®^ 80 | 32.99 | 1 | 32.99 | 43.52 | <0.0001 |
| C-Homogenisation speed | 17.70 | 1 | 17.70 | 23.34 | 0.0009 |
| AB | 3.78 | 1 | 3.78 | 4.99 | 0.0524 |
| AC | 0.2812 | 1 | 0.2812 | 0.3709 | 0.5575 |
| BC | 7.03 | 1 | 7.03 | 9.27 | 0.0139 |
| A^2^ | 240.88 | 1 | 240.88 | 317.70 | <0.0001 |
| B^2^ | 49.42 | 1 | 49.42 | 65.18 | <0.0001 |
| C^2^ | 202.04 | 1 | 202.04 | 266.47 | <0.0001 |
| Residual | 6.82 | 9 | 0.7582 | - | - |
| Lack of fit | 3.47 | 5 | 0.6942 | 0.8283 | 0.5887 |
| Pure error | 3.35 | 4 | 0.8381 | - |  |

**Table S7: Cumulative *in vitro* release (%) of DPZ from different formulations (DPZ suspension, marketed formulation, DPZ-NLCs, and optimized DPZ-PEGylated NLCs) in 0.05 M SNF (pH 6.4) containing 0.1% Tween**^®^ **80 at 37 ± 0.5 °C (mean ± SD, n = 3).**

| **Time (hrs)** | **Marketed formulation** | **DPZ Suspension** | **DPZ NLCs** | **DPZ PEGylated NLCs** |
| --- | --- | --- | --- | --- |
| 0 | 0.00 ± 0.00 | 0.00 ± 0.00 | 0.00 ± 0.00 | 0.00 ± 0.00 |
| 0.1 | 8.23 ± 1.52 | 2.01 ± 0.89 | 0.29 ± 0.08 | 0.17 ± 0.09 |
| 0.5 | 16.22 ± 0.98 | 7.87 ± 0.76 | 1.12 ± 0.22 | 0.61 ± 0.06 |
| 1 | 23.11 ± 2.13 | 9.96 ± 1.37 | 2.18 ± 0.68 | 1.12 ± 0.13 |
| 2 | 28.36 ± 1.72 | 15.84 ± 1.73 | 4.31 ± 0.87 | 2.65 ± 0.36 |
| 3 | 36.45 ± 1.74 | 25.99 ± 1.62 | 6.38 ± 0.91 | 4.1 ± 0.45 |
| 4 | 47.53 ± 2.48 | 36.93 ± 2.14 | 8.6 ± 0.69 | 4.87 ± 0.34 |
| 5 | 56.37 ± 3.21 | 43.27 ± 4.56 | 10.45 ± 0.71 | 5.21 ± 0.22 |
| 6 | 64.26 ± 3.26 | 52.88 ± 5.40 | 12.98 ± 1.52 | 6.56 ± 0.70 |
| 7 | 71.44 ± 3.92 | 61.11 ± 2.97 | 14.7 ± 2.19 | 8.1 ± 0.63 |
| 8 | 82.12 ± 2.30 | 70.98 ± 2.43 | 16.89 ± 1.70 | 9.27 ± 0.66 |
| 9 | 91.09 ± 3.21 | 84.7 ± 1.58 | 18.59 ± 3.20 | 10.87 ± 1.24 |
| 10 | 99.1 ± 2.68 | 90.6 ± 2.84 | 20.56 ± 2.69 | 11.00 ± 2.34 |
| 12 | - | 99.38 ± 2.68 | 25.12 ± 3.27 | 13.89 ± 3.03 |
| 18 | - | - | 26.12 ± 2.57 | 19.45 ± 2.82 |
| 24 | - | - | 36.31 ± 2.12 | 21.45 ± 2.74 |
| 28 | - | - | 47.26 ± 2.85 | 34.56 ± 2.53 |
| 30 | - | - | 74.12 ± 3.41 | 54.42 ± 2.71 |
| 36 | - | - | 84.12 ± 2.27 | 68.32 ± 3.43 |
| 42 | - | - | 95.13 ± 3.65 | 73.11 ± 3.01 |
| 48 | - | - | 96.42 ± 2.64 | 78.26 ± 3.16 |
| 50 | - | - | - | 84.46 ± 2.28 |
| 55 | - | - | - | 90.11 ± 1.56 |
| 58 | - | - | - | 95.61 ± 2.64 |
| 60 | - | - | - | 99.2 ± 1.98 |

**Table S8: Kinetic modelling of *in vitro* release profiles of DPZ from different formulations fitted to various mathematical models, showing correlation coefficients (R²).**

| **Formulation** | **0 order** | **1 order** | **Korsmeyer–Peppas** | **Higuchi** | **Hixon-Crowell** |
| --- | --- | --- | --- | --- | --- |
| Marketed Formulation | **0.9902** | 0.6481 | 0.5317 | 0.9843 | 0.727 |
| Drug Suspension | **0.9941** | 0.7649 | 0.7908 | 0.9302 | 0.8291 |
| DPZ NLCs | 0.9641 | 0.6818 | **0.9812** | 0.8969 | 0.837 |
| DPZ PEGylated NLCs | 0.9784 | 0.927 | **0.9869** | 0.9177 | 0.9055 |

**Table S9: *Ex vivo* cumulative diffusion (%) of DPZ across goat nasal mucosa from different formulations (DPZ suspension, DPZ-NLCs, and optimized DPZ-PEGylated NLCs) in 0.05 M SNF (pH 6.4) containing 0.1% Tween**^®^ **80 at 37 ± 0.5 °C (mean ± SD, n = 3).**

| **Time (hrs)** | **DPZ Suspension** | **DPZ NLCs** | **DPZ PEGylated NLCs** |
| --- | --- | --- | --- |
| 0 | 0.0 ± 0.0 | 0.0 ± 0.0 | 0.0 ± 0.0 |
| 0.1 | 1.53 ± 0.10 | 0.59 ± 0.21 | 0.06 ± 0.05 |
| 0.25 | 3.25 ± 0.66 | 0.92 ± 0.11 | 0.20 ± 0.04 |
| 0.4 | 4.55 ± 1.02 | 1.19 ± 0.18 | 0.33 ± 0.07 |
| 0.5 | 5.27 ± 1.30 | 1.56 ± 0.38 | 0.42 ± 0.08 |
| 0.75 | 6.37 ± 1.39 | 1.74 ± 0.45 | 0.62 ± 0.10 |
| 1 | 6.96 ± 1.82 | 2.04 ± 0.58 | 0.95 ± 0.23 |
| 2 | 12.87 ± 1.64 | 4.34 ± 1.11 | 2.40 ± 0.62 |
| 3 | 21.99 ± 2.57 | 6.14 ± 1.21 | 3.85 ± 0.82 |
| 4 | 28.27 ± 2.66 | 6.56 ± 1.33 | 4.81 ± 1.05 |
| 5 | 34.43 ± 3.31 | 7.71 ± 1.17 | 5.44 ± 1.36 |
| 6 | 48.01 ± 2.50 | 9.80 ± 1.63 | 5.97 ± 1.53 |
| 7 | 52.95 ± 3.20 | 11.17 ± 1.08 | 6.69 ± 1.46 |
| 8 | 60.33 ± 1.75 | 13.05 ± 2.12 | 8.86 ± 1.32 |
| 9 | 64.90 ± 1.93 | 15.31 ± 3.00 | 9.33 ± 1.66 |
| 10 | 72.76 ± 2.34 | 18.95 ± 1.73 | 10.61 ± 1.54 |
| 11 | 78.07 ± 2.07 | 20.25 ± 2.30 | 11.11 ± 2.34 |
| 12 | 84.75 ± 2.51 | 23.64 ± 3.33 | 12.01 ± 2.85 |
| 15 | 94.56±2.47 | 25.67 ± 3.41 | 13.45 ±2.34 |
| 24 |  | 32.49 ± 3.28 | 19.81 ±2.67 |
| 48 |  | 86.73 ±4.13 | 58.52 ±3.54 |
| 52 |  | 92.08 ±3.70 | 65.36 ±4.12 |
| 60 |  |  | 83.73 ±4.12 |
| 67 |  |  | 93.24 ±3.29 |

**List of supplementary figures:**

**Fig.S1: Particle size analysis of optimized DPZ PEGylated NLCs**

**Fig.S2:** **Particle size analysis of uncoated DPZ NLCs**

**Fig.S3:** **Zeta potential analysis of optimized DPZ PEGylated NLCs**

**Fig.S4: Zeta potential analysis of uncoated DPZ NLCs**
