# Supplementary material for: Quality by design (QbD)-guided development of PEGylated nanostructured lipid carriers of donepezil for intranasal delivery
Source: Drug Deliv Transl Res. 2026 Feb 24;16(10):3677–701. doi: 10.1007/s13346-026-02061-5 (PMC13619771; doi:10.1007/s13346-026-02061-5)
Supplement: Supplementary file 2 [file 13346_2026_2061_MOESM2_ESM.docx]

**Analytical Method Validation (Spectrofluorimetric Assay)**

A spectrofluorimetric method was developed and validated for the quantification of Donepezil (DPZ) in simulated nasal fluid (SNF, 0.05 M, pH 6.4) containing 0.1% Tween® 80. A stock solution of DPZ (1 mg/mL) was prepared in SNF with 0.1% Tween® 80 and diluted to prepare working standards in the range of 10–100 ng/mL. Fluorescence measurements were performed using a Shimadzu RF-5301 PC spectrofluorometer (Japan) at an excitation wavelength of 314 nm and an emission wavelength of 628 nm, with slit widths fixed at 5 nm. Each concentration was analysed in triplicate and the average values were used to generate the calibration plot. The method demonstrated excellent linearity across the range of 10–100 ng/mL with a correlation coefficient (R²) of 0.9994. The limit of detection (LOD) and limit of quantification (LOQ) were determined using the standard deviation of the blank response and the slope of the calibration curve, calculated as 3.3σ/S and 10σ/S, respectively. Intra-day and inter-day precision were evaluated at three different concentrations (20, 60, and 100 ng/mL) and the relative standard deviation remained below 2% in all cases, confirming good repeatability. Accuracy was assessed by recovery studies performed at 80%, 100%, and 120% of nominal concentrations, and the recoveries ranged between 98% and 102%. Specificity of the method was confirmed by analysing blank SNF and placebo NLC formulations, both of which showed no measurable interference at the analytical wavelengths. On the basis of these results, the developed method was considered sensitive, accurate, and selective for the estimation of DPZ in in vitro and ex vivo release samples. The calibration data are provided in Table 1 and a representative calibration curve is shown in Figure 1.

**Table 1.** Calibration data for Donepezil in 0.05 M simulated nasal fluid (pH 6.4) containing 0.1% Tween® 80, determined by the spectrofluorimetric method (mean ± SD, n = 5).

| **Concentration**  **(ng/ml)** | **Intensity**  **(1)** | **Intensity**  **(2)** | **Intensity**  **(3)** | **Intensity**  **(4)** | **Intensity**  **(5)** | **Mean ± SD** |
| --- | --- | --- | --- | --- | --- | --- |
| 10 | 208 | 195 | 205 | 198 | 190 | 199.2 ± 7.32 |
| 20 | 256 | 272 | 285 | 276 | 280 | 273.8± 11.05 |
| 40 | 367 | 420 | 366 | 400 | 380 | 386.6± 23.16 |
| 60 | 491 | 494 | 495 | 504 | 520 | 500.8± 11.77 |
| 80 | 621 | 639 | 623 | 595 | 630 | 621.6± 16.45 |
| 100 | 728 | 790 | 778 | 714 | 729 | 747.6± 33.98 |

CC of DPZ in 0.1% Tween 80 + 0.05M SNF 6.4

Intensity

| Concentration (ng/ml) | AVERAGE |
| --- | --- |
| 10 | 199.2 |
| 20 | 273.8 |
| 40 | 386.6 |
| 60 | 500.8 |
| 80 | 621.6 |
| 100 | 747.6 |

**Figure 1.** Calibration curve of Donepezil in 0.05 M simulated nasal fluid (pH 6.4) containing 0.1% Tween® 80, obtained by the spectrofluorimetric method.
